# Taxifolin alleviates podocyte injury and macrophage chemotaxis associated with diabetic nephropathy by directly targeting RBP1 identified via multi-omics and machine learning

**DOI:** 10.3389/fphar.2026.1933167

**Published:** 2026-10-12

**Authors:** Zi-Xuan Wang, Wen-Xuan Wang, Qiong-Dan Liang, Ling-Dong Kong

**Affiliations:** State Key Laboratory of Pharmaceutical Biotechnology, Department of Pharmacy, Nanjing Drum Tower Hospital, School of Life Sciences, Nanjing University, Nanjing, China

**Keywords:** diabetic nephropathy, Linggui Zhugan formula, machine learning, multi-omics, podocyte injury, RBP1, taxifolin

## Abstract

**Aims:**

Podocyte injury contributes to diabetic nephropathy (DN) progression. This study aimed to screen and validate the key functional proteins related to podocyte injury in DN, and to explore the protective effect of bioactive ingredients derived from traditional Chinese medicine Linggui Zhugan (LGZG) formula against DN.

**Methods:**

Proteomics, weighted gene co-expression network analysis, machine learning, and repeated out-of-fold exact SHapley Additive exPlanations (TreeSHAP) analysis were integrated to screen the top 15 core differentially expressed proteins in kidney tissues of DN patients. Single-cell transcriptomics and cell-cell communication analysis were further used to identify the podocyte protein retinol-binding protein 1 (RBP1). *In vitro*, a palmitic acid (PA)-induced podocyte injury model and gene knockdown experiments were performed to verify the regulatory function of RBP1. Molecular docking and microscale thermophoresis (MST) were applied to screen and confirm the binding interaction between bioactive ingredients of LGZG formula and RBP1. The renoprotective effect of taxifolin was further evaluated in this cell model.

**Results:**

Integrative multi-omics and machine learning identified RBP1 as a key pathogenic molecule in DN patients. *In vitro*, PA stimulation increased RBP1 protein expression in podocyte injury, with the downregulation of podocyte-specific slit diaphragm proteins nephrin and synaptopodin, and the upregulation of pro-inflammatory mediators including C-C motif chemokine ligand 2 (*CCL2*), interleukin-6 (*IL-6*), and tumor necrosis factor-α (*TNF-α*), as well as enhanced macrophage chemotaxis. Notably, *RBP1* knockdown reversed these PA-induced alterations to alleviate podocyte injury. Taxifolin from LGZG formula directly bound RBP1, as confirmed by molecular docking and MST. Furthermore, taxifolin was found to bind to RBP1. And then it suppressed nuclear factor κB (NF-κB) signaling pathway, reduced *CCL2*, *IL-6* and *TNF-α* mRNA expression, inhibited macrophage chemotaxis, and upregulated nephrin and synaptopodin expression, thereby ameliorating PA-induced podocyte injury.

**Conclusion:**

This study identified RBP1 as a novel driver of podocyte injury and macrophage chemotaxis associated with DN via multi-omics and machine learning. Taxifolin, a bioactive compound from the LGZG formula, directly bound to RBP1 and alleviated podocyte injury. These findings highlight RBP1 as a promising therapeutic target and support the potential of taxifolin as a targeted intervention for DN progression.

## 1 Introduction

Diabetic nephropathy (DN), a major complication of diabetes mellitus, has emerged as the leading cause of end-stage renal disease (ESRD) worldwide (Sun et al., 2022; Zheng et al., 2018). DN is characterized by a decreased glomerular filtration, albuminuria, accumulation of mesangial matrix, and aggravation of podocyte injury (Tervaert et al., 2010; Xu et al., 2026). Podocytes are terminally differentiated epithelial cells and form the key structure of the glomerular filtration barrier. Destruction of podocyte architecture causes barrier dysfunction and proteinuria (Li and Liu, 2025; Zhao et al., 2024; Reidy et al., 2014). Macrophage infiltration and podocyte inflammation aggravate podocyte injury (Li et al., 2022). Blocking macrophage infiltration and suppressing C-C motif chemokine ligand 2 (CCL2) and C-C chemokine receptor 2 (CCR2) effectively alleviates renal injury in streptozotocin (STZ)-induced DN mouse model (Awad et al., 2011; Boels et al., 2017). Thus, the identification of molecular targets that drive podocyte injury and macrophage chemotaxis may be essential for the discovery of new therapies against progressive DN.

Retinol-binding protein 1 (RBP1) encodes cellular retinol-binding protein 1 (CRBP1), a 15 kDa cytosolic retinol-binding protein that regulates intracellular retinol handling and vitamin A homeostasis (Ghyselinck et al., 1999; Napoli, 2017). Of note, RBP1 expression is altered in lipid-rich metabolic states: hepatic RBP1 expression is increased in human non-alcoholic fatty liver disease (NAFLD) (Ashla et al., 2010), whereas high-fat-diet-induced obesity and increasing hepatic steatosis are associated with reduced Crbp1 expression and impaired tissue vitamin A signaling (Trasino et al., 2015). Dysregulated lipid metabolism and renal lipid accumulation are prominent features of DN (Herman-Edelstein et al., 2014; Wang et al., 2024), while elevated circulating Free Fatty Acids (FFAs) are closely linked to diabetes-associated metabolic dysfunction (Bergman and Ader, 2000). Among FFAs, palmitic acid (PA), the most abundant saturated fatty acid in human and rodent plasma, is markedly elevated in diabetes, as well as in DN (Bergman and Ader, 2000; Richieri and Kleinfeld, 1995; Wang et al., 2005). Lipid overload promotes podocyte injury in DN patients (Herman-Edelstein et al., 2014). Elevated PA levels contribute to renal lipotoxicity, leading to podocyte dysfunction, oxidative stress, and apoptosis in PA-induced DN mice (Lee et al., 2021). PA-induced podocyte injury has been widely used as an *in vitro* model to mimic renal lipotoxicity in DN (Mori et al., 2021; Qin et al., 2019). However, whether RBP1 participates in PA-induced podocyte injury associated with DN progression remains unclear.

Linggui Zhugan (LGZG) formula, first recorded in Zhang Zhongjing’s Jin Gui Yao Lue (Essential Prescriptions of the Golden Cabinet), is a classical traditional Chinese medicine formula comprising Poria, *Cinnamomi ramulus*, *Atractylodis macrocephalae* rhizome, and Glycyrrhizae radix et rhizome. Clinically, this classical formula has long been utilized for the management of diverse disorders caused by Yang deficiency (Chen et al., 2012). LGZG formula significantly attenuates DN-like traits and metabolic dysfunction in high-fat diet/STZ-induced DN mice (Yi et al., 2024). However, due to its complex chemical composition, the pharmacological material basis and specific therapeutic targets of LGZG formula against DN remain largely unclear.

Target-based virtual screening is a widely adopted strategy for identifying bioactive ingredients from traditional Chinese medicine formulas. Using RBP1 as the target, we screened major active components of LGZG formula via molecular docking and microscale thermophoresis (MST) validation, and identified taxifolin as the ligand with the strongest binding affinity. Taxifolin is reported to alleviate hyperglycemia and hyperuricemia, improve renal function, showing the therapeutic potential in STZ-induced DN rats (Zhao et al., 2018). However, whether taxifolin targets RBP1 to ameliorate podocyte injury in DN remains unclear.

This study combines kidney proteomics, weighted gene co-expression network analysis (WGCNA), a single regularized XGBoost framework with repeated out-of-fold exact TreeSHAP feature attribution, and single-cell transcriptomics to prioritize DN-associated proteins and identify RBP1 as a podocyte-associated pathogenic candidate. The overall study design and analytical workflow are illustrated in Figure 1. Experiments *in vitro* demonstrated that PA induced the upregulation of podocyte RBP1 protein expression, leading to podocyte injury and macrophage chemotaxis. Taxifolin derived from the LGZG formula may directly bind to RBP1 to alleviate podocyte injury. These findings identify a protein target that drives podocyte injury in DN and provide a potential therapeutic natural compound.

**FIGURE 1:**
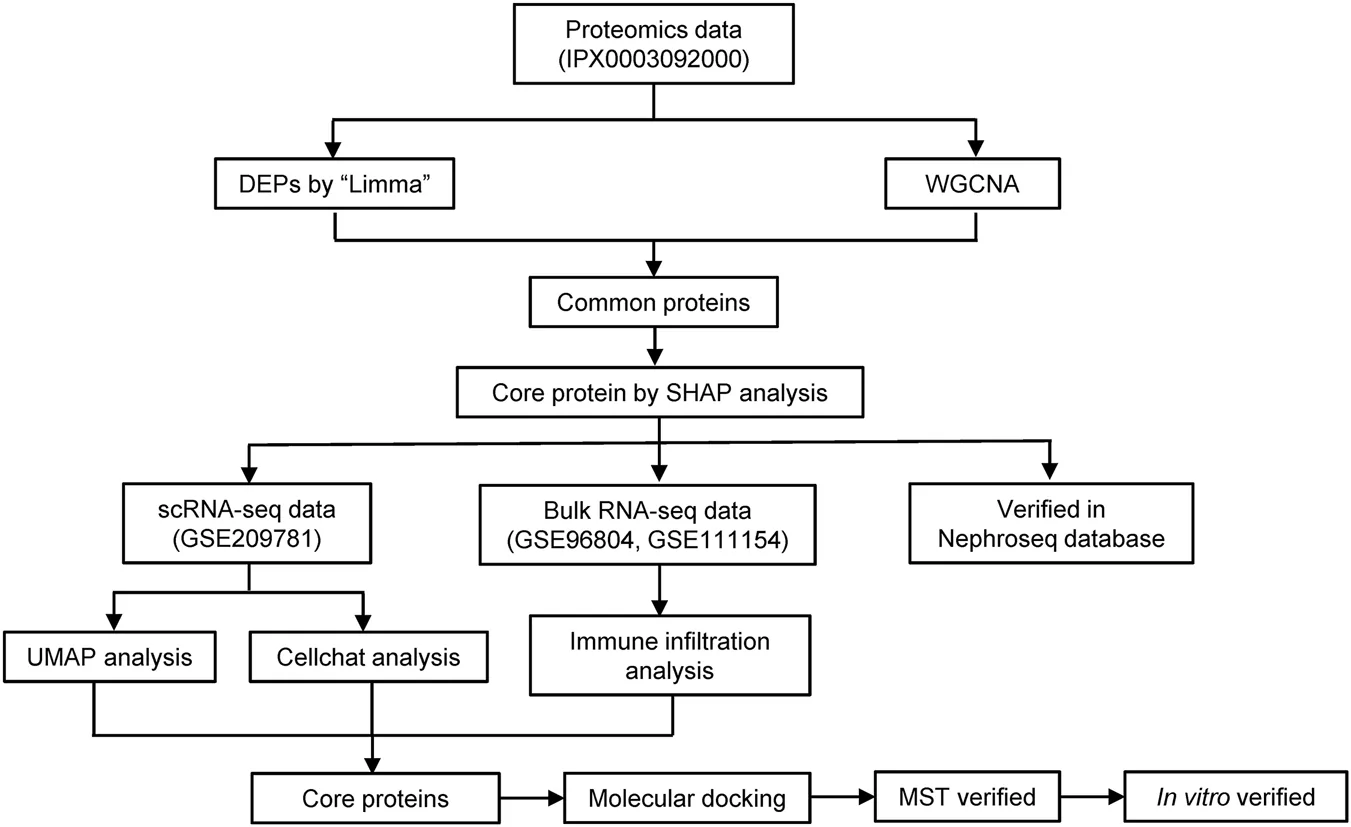
Flow diagram. Starting from the proteomics dataset (IPX0003092000), DEPs were screened using the limma algorithm, and a protein co-expression network was concurrently constructed via WGCNA. After extracting the overlapping proteins from the intersection of these two analytical approaches, core proteins were identified through integration with SHAP interpretable analysis. For these core proteins, multi-omics validation and functional exploration were conducted across three complementary directions: (1) scRNA-seq data (GSE209781) was integrated to characterize the cell-type-specific distribution of core proteins via UMAP dimensionality reduction analysis, and intercellular communication networks in DN were deciphered using CellChat analysis; (2) Bulk RNA-seq datasets (GSE96804 and GSE111154) were used to profile the immune cell infiltration landscape in DN renal tissues; (3) The clinical relevance of core proteins was validated using the Nephroseq database. Results from the above multi-omics analyses converged to identify the key protein RBP1. Subsequently, potential natural compounds targeting RBP1 were predicted via molecular docking, and direct binding affinity between the candidate compound and RBP1 was verified using microscale thermophoresis (MST) assays. Finally, the pathogenic role of RBP1 and the protective effect of its targeting compound were validated using *in vitro* podocyte injury models.

## 2 Materials and methods

### 2.1 Data source

Label-free quantitative proteomics data were obtained from the ProteomeXchange (proteomexchange.org) repository under accession number IPX0003092000. Protein abundance data were log2-transformed and normalized using median centering. For single-cell RNA sequencing (scRNA-seq), the dataset GSE209781 was downloaded, which contains kidney tissue from three control and 3 DN patients. Raw read counts were normalized using the trimmed mean of M-values (TMM) method implemented in the “edgeR” package. Batch effects were adjusted using the ComBat algorithm from the “sva” package.

### 2.2 Differential expression analysis

Differential expression analysis was performed using the “limma” package (v3.50.0) in R. Genes and proteins with an P < 0.05 and |log2FC| ≥ 2 were considered statistically significant.

A variance-based filtering step was applied prior to dimensionality reduction analysis. Proteins ranking in the bottom 20% by variance across all samples were removed, and the top 80% most variable proteins were retained for all downstream multivariate analyses.

Principal component analysis (PCA) was performed using the “prcomp” function in R to visualize the overall protein expression profiles of DN and control kidney tissues. Permutational multivariate analysis of variance (PERMANOVA) was conducted based on Euclidean distance with 999 permutations using the “vegan” package (v2.6-4) in R, to statistically test the significance of overall proteomic differences between the two groups.

### 2.3 WGCNA

The input data for WGCNA were derived from the quality-filtered renal proteomic expression matrix. Briefly, raw protein intensity data were first log2-transformed and then normalized via quantile normalization using the “limma” package. After variance-based filtering, the top 80% most variable proteins were retained to reduce background noise and improve the specificity of co-expression module detection. The normalized and filtered expression matrix (with samples in rows and proteins in columns) was used for subsequent WGCNA analysis.

WGCNA was carried out using the WGCNA package (v1.70) in R. A soft-thresholding power of 3 was selected to achieve a scale-free topology fit index ≥0.9. Co-expression modules were identified using the dynamic tree cut algorithm (minModuleSize = 30, mergeCutHeight = 0.25). Module eigengenes (MEs) were calculated and correlated with clinical traits of DN. The turquoise module, which showed the highest positive correlation with DN, was selected for downstream analysis.

### 2.4 Machine learning-based feature selection

To prioritize the candidate proteins, a single regularized XGBoost classifier was used. Samples were stratified into a training set (70%) and a held-out test set (30%), with the test set used only for final performance evaluation. Within the training set, hyperparameters governing model complexity and regularization, including tree depth, learning rate, row/feature subsampling, L1/L2 penalties, and split constraints, were optimized using repeated 5-fold cross-validation with three repeats. Model discrimination was evaluated on the held-out test set using ROC analysis with DeLong 95% CIs, together with confusion matrices and standard classification metrics.

Feature attribution was performed using exact XGBoost TreeSHAP within a repeated out-of-fold framework. For each fold, SHAP values were calculated only for samples not used to train that fold model, and mean absolute out-of-fold TreeSHAP values across repeated 5-fold cross-validation were used to rank proteins. The 15 highest-ranking proteins with non-zero contributions were selected for downstream analyses.

### 2.5 Single-cell RNA sequencing analysis

ScRNA-seq data were processed using Seurat (v4.1.0). Low-quality cells were filtered out (genes <200 or >6,000; mitochondrial gene percentage >10%). Data normalization was performed using SCTransform. Dimensionality reduction was conducted using PCA followed by Uniform Manifold Approximation and Projection (UMAP). Cell clusters were identified using the FindClusters function (resolution = 0.8). Differentially expressed marker genes for each cluster were identified using the FindAllMarkers function, with thresholds set at log2FC > 1, minimum percentage of expressing cells (min.pct) > 0.2, and P < 0.05. Cell types were assigned manually by integrating cluster-specific marker genes with well-recognized canonical markers of kidney and immune cell populations (Supplementary Table S1).

### 2.6 Cell-cell interaction analysis

Intercellular communication networks were constructed using CellChat (v1.1.3). Ligand–receptor interactions were inferred from the CellChatDB database. Communication probability was calculated using the trimean-based method. Differential network analysis between control and DN groups was performed using the compareInteractions function.

### 2.7 Materials and reagents

Palmitic acid powder (P0500, Sigma-Aldrich) was dissolved in 0.1 M NaOH solution preheated to 75 °C to form a 40 mM sodium palmitate saponification solution. Meanwhile, a 40% (w/v) fatty acid-free bovine serum albumin (BSA) solution was prepared in phosphate-buffered saline (PBS) and prewarmed in a 55 °C water bath. An equal volume of the prewarmed BSA solution was slowly added to the sodium palmitate solution maintained at 75 °C, and the mixture was thoroughly homogenized to obtain a 20 mM PA-BSA stock solution. The stock solution was filter-sterilized through a 0.22-μm membrane, aliquoted, and stored at −20 °C in the dark. Before use, an appropriate aliquot was thawed and completely reconstituted in a 55 °C water bath, then diluted with serum-free medium to the desired working concentration.

Taxifolin (PHL89284, Sigma-Aldrich) was dissolved in DMSO, with the volume of solvent added directly based on the target concentration.

### 2.8 Cell culture, treatment, and transfection

The conditionally immortalized human podocyte cell line (HPCs) was a generous gift from Dr. Zhi-Hong Liu (Research Institute of Nephrology, Nanjing General Hospital of Nanjing Military Command, Nanjing, Jiangsu Province, P. R. China), which was provided by M. Saleem (University of Bristol, Bristol, United Kingdom). Mouse podocyte clone-5 (MPC5) cells were obtained from Shanghai Fuheng Biotechnology Co., Ltd. HPCs and MPC5 cells were cultured using a consistent temperature-dependent proliferation-differentiation protocol. For proliferation, both cell lines were maintained at 33 °C in a 5% CO_2_ atmosphere in Roswell Park Memorial Institute (RPMI) 1640 medium supplemented with 10% fetal bovine serum (FBS), 1% penicillin-streptomycin, and 10 U/mL recombinant interferon-γ (IFN-γ). To induce differentiation, cells were shifted to 37 °C and cultured in the same basal medium without IFN-γ for 7 days. Differentiated podocytes were used for all subsequent experiments.

A human monocyte leukemia cell line (THP-1) was purchased from Wuhan Pricella Biotechnology Ltd (CL-0233, Servicebio, Wuhan, China). The cells were cultured at 37 °C with 5% CO_2_ in RPMI 1640 medium supplemented with 10% FBS and 100 U/mL penicillin. In all experiments, THP-1 monocytes were treated with 100 nM phorbol 12-myristate 13-acetate (PMA) (P1585, Sigma-Aldrich) for 24 h to transform into M0 macrophages.

THP-1 monocytes were differentiated into M0 macrophages with 100 nM PMA for 24 h. To assess the effect of podocyte *RBP1* on macrophage polarization, differentiated HPCs transfected with siRNA-NC or siRNA-*RBP1* were treated with fatty acid-free BSA or 100 μM PA for 24 h. The medium was then removed, cells were gently washed with PBS, and fresh PA-free differentiation medium (RPMI 1640 containing 10% FBS and 1% penicillin-streptomycin, without IFN-γ) was added for 16 h to collect conditioned medium (CM). The same procedure was applied to all groups. Four CM groups were generated: (1) siNC-CM, CM from BSA-treated HPCs transfected with siRNA-NC; (2) PA + siNC-CM, CM from PA-treated HPCs transfected with siRNA-NC; and (3) PA + si*RBP1*-CM, CM from PA-treated HPCs transfected with siRNA-*RBP1*; (4) si*RBP1*-CM, CM from BSA-treated HPCs transfected with siRNA-*RBP1*.

After PMA-induced differentiation, the THP-1 culture medium was removed and replaced with the corresponding HPC-derived CM for 24 h without additional PA or PMA supplementation. CM from BSA-treated siNC-transfected HPCs was used as the baseline control because it accounted for the basal podocyte secretome, siRNA transfection, and CM preparation procedure, thereby allowing the effects of PA stimulation and *RBP1* knockdown to be evaluated under matched conditions.

The mouse macrophage cell line RAW264.7 was purchased from Wuhan Pricella Biotechnology Ltd (CL-0190, Servicebio, Wuhan, China). RAW264.7 cells were cultured in Dulbecco’s Modified Eagle Medium (DMEM) supplemented with 10% fetal bovine serum, 100 U/mL penicillin, and 0.1 mg/mL streptomycin at 37 °C with 5% CO_2_.

After treatment with PA for 0, 12, 24 or 36 h, HPCs and MPC5 cells were analyzed in various experiments as described below. For drug intervention, cells were treated with or without PA (100 μM), taxifolin (5, 10, and 20 μM), dapagliflozin (5 μM, SML2804, Sigma-Aldrich) for 24 h, respectively.

For transfection, Lipofectamine 2000 reagent (11,668-019, Invitrogen) was used to deliver short interfering RNA (siRNA) into cells following the manufacturer’s instructions. (1) HPCs were transfected with siRNA-Negative control (siRNA-NC) or siRNA-*RBP1*, subsequently stimulated with 100 μM PA or not. (2) MPC5 cells were transfected with siRNA-NC or siRNA-*Rbp1*, subsequently stimulated with 100 μM PA or not. human-*RBP1* siRNA forward: GGAAGAUGUUGGUCAACGAGAdTdT, reverse: UCG​UUG​ACC​AAC​AUC​UUC​CAG dTdT; mouse-*Rbp1* siRNA forward: GGAGCUGAAGCUAGUAAUAGAdTdT, reverse: UAUUACUAGCUUCAGCUCCUAdTdT; Negative control forward: UUCUCCGAACGUGUCACGUdTdT, reverse: ACGUGACACGUUCGGAGAAdTdT.

### 2.9 Cell viability assay

Cell viability was determined using the Cell Counting Kit-8 (CCK-8) assay. Briefly, differentiated HPCs or MPC5 cells were seeded into 96-well plates at a density of 5 × 10^3^ cells per well overnight. For concentration-gradient analysis, cells were treated with serial concentrations of PA (50, 100, 200, 300, and 500 μM) or an equivalent volume of BSA vehicle control for 24 h. For time-course analysis, cells were incubated with 100 μM PA for 0, 12, 24, 36, 48, and 72 h, respectively. After treatment, 10 μL of CCK-8 reagent was added to each well, and the plates were incubated at 37 °C for 2 h in the dark. The absorbance at 450 nm was measured using a microplate reader.

### 2.10 Western blot analysis

Protein extracts from HPCs and MPC5 cells were homogenized in lysis buffer, followed by centrifugation at 12, 000 *g* for 15 min at 4 °C, respectively. Proteins in equal quantities from every sample were subjected to electrophoresis on 10% SDS-PAGE gels and electrotransferred to polyvinylidene fluoride membranes (IPVH100010, Millipore). Following a blocking step, the membranes probed with primary antibodies and secondary antibodies. Anti-nephrin (22,912-1, 1:2,000, proteintech); anti-synaptopodin (21,064-1, 1:3,000, proteintech); anti-RBP1(22,683-1, 1:2,000, proteintech); anti-p-p65 (ab76302, 1:2,000, Abcam); anti-p65 (ab32536, 1:2,000, Abcam); anti-IκBα (ab32518, 1:2,000, Abcam). Densitometry was used to quantify the immunoreactive blots using ImageJ software, standardizing the data to β-actin, expressing the data as fold changes in relation to control.

### 2.11 RNA extraction and quantitative real-time (qRT)-PCR

RNA isolation of HPCs and MPC5 cells was conducted using the Trizol method (R401-01-AA, Vazyme), as recommended by the manufacturer, respectively. 1 μg of total RNA was produced with HiScript® II Q RT SuperMix (R222, Vazyme) to synthesize single-strand cDNA. Complementary DNA was amplified using ChamQTM SYBR® qPCR Master Mix (Q311, Vazyme) in a Bio-Rad PCR System. The mRNAs were normalized to β-actin. The primer sequences were shown in the Supplementary Table S2.

### 2.12 Transwell assay

For the THP-1 macrophage chemotaxis assay, THP-1 monocytes were first induced to differentiate into adherent macrophages with 100 nM PMA for 24 h. The differentiated cells were digested, resuspended in serum-free RPMI 1640 medium, and 100 µL of the cell suspension was seeded into the upper Transwell chamber (8-µm wells, 24-well plate). The lower chamber was filled with 700 µL of conditioned medium from differently treated HPCs (diluted in serum-free RPMI 1640 medium). The cells remaining on the top surface of the polycarbonate insert were gently removed with a cotton swab after 24 h of incubation. The cells moved to the lower membrane were fixed with 4% paraformaldehyde for 15 min and stained with 0.1% crystal violet for 15 min at room temperature.

For the RAW264.7 macrophage chemotaxis assay, 100 µL of a RAW264.7 cell suspension was inoculated in an upper Transwell chamber (8-µm wells, 24-well plate), followed by the addition of serum-free DMEM 700 µL conditioned medium of MPC5 after different treatments in the lower chamber. After 24 h of incubation, non-migrated cells on the upper membrane surface were wiped off with a cotton swab, and the migrated cells on the lower surface were fixed and stained following the same protocol as described for THP-1 cells.

### 2.13 Co-immunoprecipitation assay

Co-immunoprecipitation (Co-IP) was performed to examine the interaction between IκBα and p65. HPCs subjected to the indicated treatments were lysed in ice-cold IP lysis buffer containing protease and phosphatase inhibitors. Equal amounts of total protein were incubated with anti-p65 antibody overnight at 4 °C, followed by incubation with protein A/G agarose beads. Normal IgG was used as a negative control. The immunoprecipitated complexes were washed, eluted, and subjected to Western blot analysis using antibodies against IκBα and p65. Aliquots of whole-cell lysates were used as input controls to assess total IκBα and p65 levels.

### 2.14 Immunofluorescence assay

The differentiated HPCs were washed with cold PBS, then fixed with 4% paraformaldehyde for 20 min at room temperature. These cells were permeabilized with 0.2% Triton X-100 for 5 min after washing with PBS (3 times), then blocked with immunostaining blocking buffer for 1 h. The podocytes were labeled with primary antibody mouse to p65 (dilution 1:50) at 4 °C overnight. The expression levels of p65 were detected with Alexa Fluor 488 goat anti-mouse (dilution 1:500) secondary antibody. The nucleus was counterstained with DAPI and coverslipped with aqueous mounting medium. Finally, all samples were examined with a confocal laser scanning microscope (Leica TCS SP8-MaiTai M, Germany).

### 2.15 MST assay

MST instrument Monolith NT.115 (NanoTemper Technologies, Munich) was used to conduct the binding assay of stellasterol, glycyrol, taxifolin and RBP1, respectively. RBP1-eGFP cell lysates were ensured that the fluorescence intensity of RBP1 during the MST assay was about 500RU. Taxifolin, stellasterol and glycyrol (10 mM) were serially diluted 16 times with MST buffer (containing 0.05% Tween-20), respectively. Each dilution was mixed 1:1 with the RBP1-eGFP lysate and incubated for 10 min at room temperature in the dark. The binding reaction was then loaded into standard glass capillaries, and MST measurement was performed on the NT.115 instrument following the manufacturer’s protocol.

### 2.16 Molecular docking

Molecular docking was performed to evaluate binding interactions between candidate compounds and core gene-encoded proteins. Protein structures were obtained from the RCSB Protein Data Bank (PDB). Docking simulations were conducted using cbdock2 (http://cao.labshare.cn:11080/cb-dock2/php/blinddock.php). Docking poses with binding energy ≤ −5.0 kcal/mol were considered stable. Results were visualized using Pymol.

### 2.17 Validation in nephroseq database

The expression and clinical relevance of core genes were further validated using the Nephroseq v5 database (https://www.nephroseq.org). Datasets with glomerular filtration rate (GFR) or serum creatinine levels were selected. Significance was set at P < 0.05.

### 2.18 Statistical analysis

All statistical analyses were performed in R (v4.2.0). Continuous variables were expressed as mean ± standard deviation. Group comparisons were conducted using Student’s t-test or One-way ANOVA test. P < 0.05 was considered statistically significant.

## 3 Results

### 3.1 Identification of potential core proteins in DN patients by integrating differential expression analysis and WGCNA

To characterize the differences in the kidney proteome between DN patients and controls, we first evaluated the overall protein expression profiles between DN and control samples via PCA. The results showed that Principal component (PC) 1 explained 17.8% of the total variance and PC2 explained 7.5% of the variance, with a cumulative variance explanation rate of 25.3% (Figure 2A). PERMANOVA further demonstrated a significant difference in the overall proteomic profiles between the DN and control groups (R^2^ = 0.1576, P = 0.001), with disease status accounting for approximately 15.8% of the total proteomic variance (Supplementary Table S3). PC1 was mainly contributed by glomerular and tubular injury-related molecules (such as Low-density lipoprotein receptor-related protein 2 (LRP2), Phospholipase A2 receptor 1 (PLA2R1)). PC2 was loaded with proteins enriched in cell cycle and RNA metabolism (such as Insulin-like growth factor 2 mRNA-binding protein 2 (IGF2BP2), DNA topoisomerase II alpha (TOP2A)) (Supplementary Figure S1).

**FIGURE 2:**
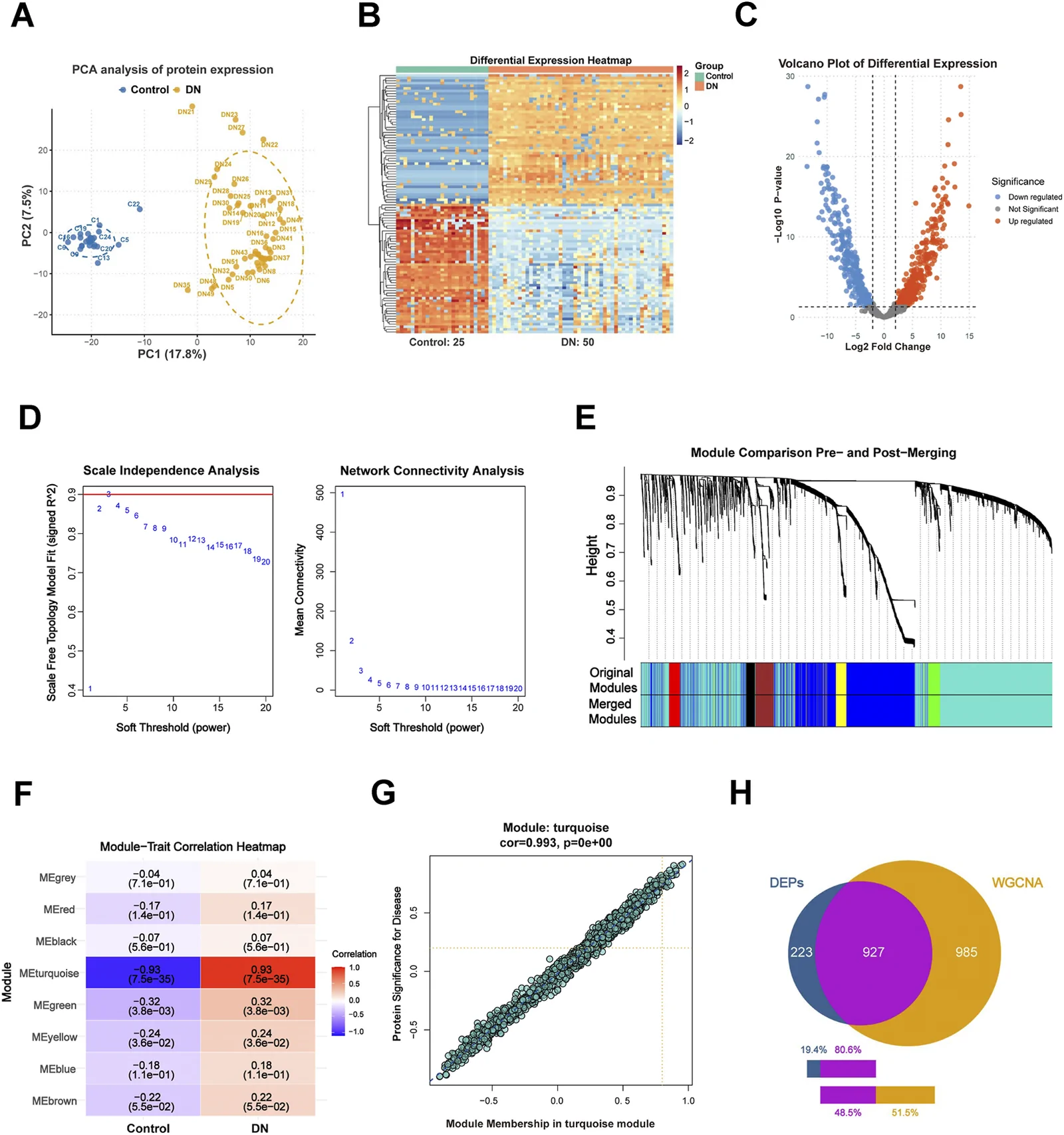
Identification of differential proteins in patients with DN and WGCNA. **(A)** Principal component analysis (PCA) of protein expression. The plot depicts the distribution of control (blue) and diabetic nephropathy (orange) samples in the PCA space. **(B)** Differential expression heatmap of IPX0003092000. **(C)** Volcano plot of differential expression. **(D)** Scale independence analysis (left) and network connectivity analysis (right) for determining the soft threshold in WGCNA. **(E)** Module comparison pre- and post-merging. The top plot shows the height of module merging, and the bottom plot shows original modules (upper colored bars) and merged modules (lower colored bars). **(F)** Module–trait correlation heatmap. Color intensity and color key indicate the strength and direction of correlation. **(G)** Scatter plot of protein significance for disease vs. module membership in the turquoise module; **(H)** Venn diagram showing the overlap between DEPs and proteins identified by WGCNA.

Subsequently, differential expression analysis was performed between DN patients and controls. Hierarchical clustering was performed on the differentially expressed proteins (DEPs), and a heatmap was generated. The protein expression patterns of kidney from the control group and DN patients showed clear clustering. The trends of upregulated or downregulated protein expression in DN patients were consistent and significantly different from those in the control group (Figure 2B). Using |log2FC| ≥ 2 and P < 0.05 as thresholds, a total of 1,150 DEPs were identified (Figure 2C).

To explore protein co-expression patterns in DN, we performed WGCNA. We first determined the optimal soft threshold to ensure the network satisfied the scale-free topology criterion, with scale independence and network connectivity analyses presented (Figure 2D). After systematic evaluation of network topology across a series of soft-thresholding powers, a soft-thresholding power of three was selected. At this threshold, the scale-free topology model fit reached ≥0.9 and the network maintained appropriate mean connectivity, confirming that the constructed network conformed to the scale-free property without excessive loss of effective inter-protein connections. Subsequently, we conducted module detection and merging, with comparisons of module profiles before and after merging illustrated (Figure 2E). The dynamic tree-cutting algorithm initially identified multiple modules, and modules with highly similar expression patterns were merged to generate a set of final co-expression modules for subsequent analyses. To identify modules closely associated with DN, we analyzed the correlation between module eigengenes and DN traits, with the resulting module-trait correlation heatmap (Figure 2F). Each cell in the heatmap denotes the correlation coefficient and P-value between a module and a trait. Notably, the MEturquoise module exhibited a strong positive correlation with DN, suggesting that proteins within this module likely play pivotal roles in DN. For further validation of the MEturquoise module, we plotted the relationship between module membership and protein significance for DN (Figure 2G). The turquoise module was highly correlated with protein significance for DN, with a correlation coefficient of 0.993 (P < 0.01). This strong correlation indicated that proteins in the turquoise module were not only co-expressed but also significantly associated with DN, confirming it as a key module involved in DN progression.

To refine candidate proteins for DN, we intersected DEPs with proteins in the WGCNA-identified modules, and a Venn diagram illustrating this intersection was shown in Figure 2H. A total of 927 proteins overlapped between DEPs and WGCNA modules; these overlapping proteins were potential core proteins in DN, as they were both differentially expressed and involved in co-expression networks.

### 3.2 Machine learning-driven feature prioritization and functional enrichment analysis in DN

A single regularized XGBoost model was used to prioritize DN-associated candidate proteins. The model achieved an AUC of 0.981 (95% CI: 0.937–1.000) (Figure 3A). The corresponding confusion matrix and classification metrics are provided in Supplementary Figure S2 and Supplementary Table S4. In the independent GSE96804 cohort, the genes corresponding to the 15 prioritized proteins showed variable single-gene discriminatory performance, with AUC values ranging from approximately 0.529 to 0.971 (Figure 3E). These findings indicated that the prioritized proteins differed in their individual discriminatory ability.

**FIGURE 3:**
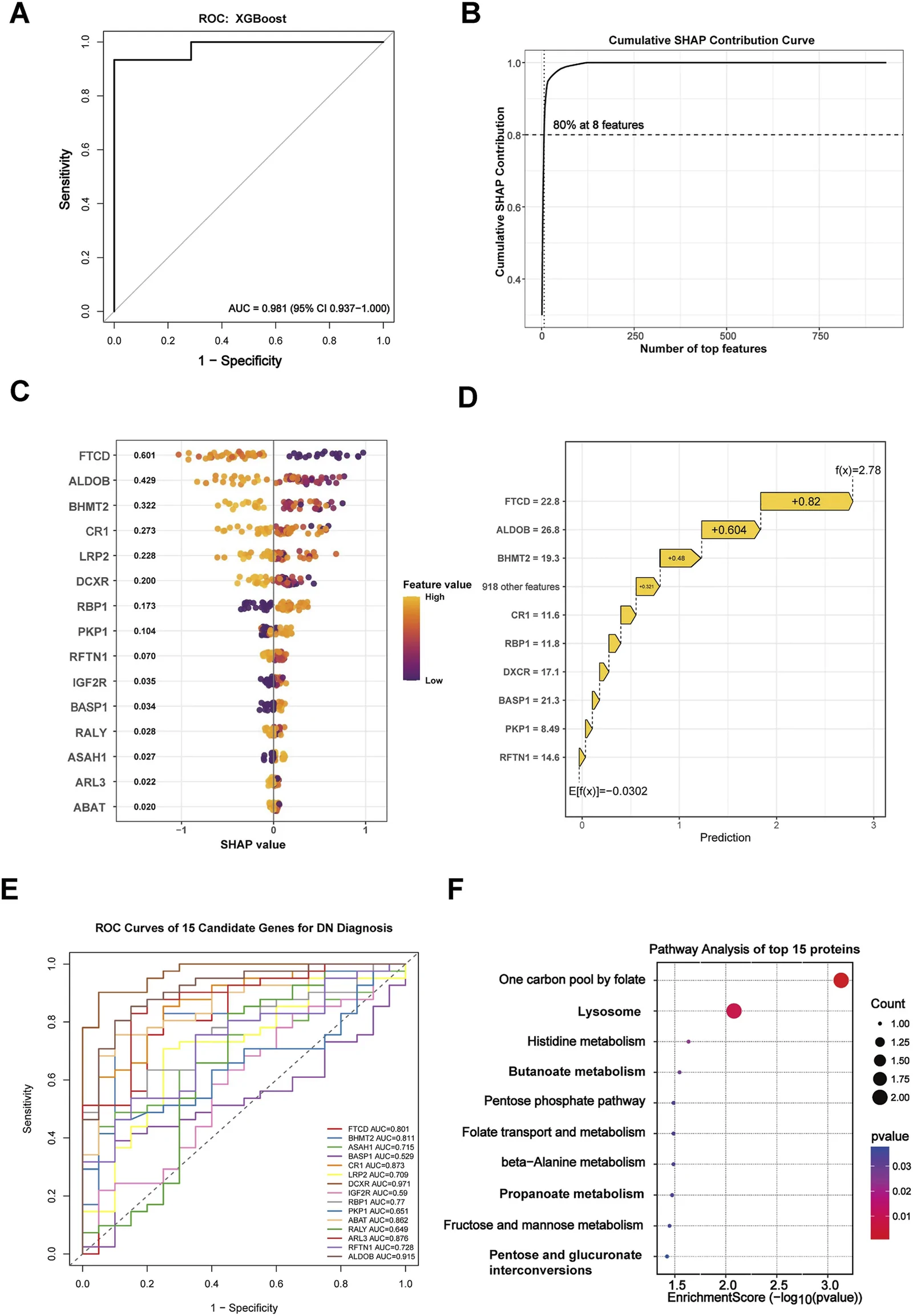
Machine learning model performance, SHAP-based feature interpretation, and functional enrichment analysis. **(A)** Receiver operating characteristic (ROC) curve of the regularized XGBoost model. **(B)** Cumulative mean absolute TreeSHAP contribution curve. **(C)** Repeated out-of-fold TreeSHAP beeswarm plot for the Top15 prioritized proteins. Each point represents a sample-level SHAP contribution; color denotes the relative feature value. **(D)** Representative TreeSHAP waterfall plot showing the local decomposition of the XGBoost output from the baseline E[f(x)] to the final prediction f(x) for one sample. **(E)** ROC curves of the genes corresponding to the 15 prioritized proteins in an independent transcriptomic cohort for DN discrimination. **(F)** KEGG pathway enrichment analysis of the 15 prioritized proteins.

Feature contributions were further quantified using repeated out-of-fold exact TreeSHAP. The cumulative contribution curve showed that the top eight features accounted for 80% of the total mean absolute SHAP contribution (Figure 3B), indicating that the predictive information was mainly concentrated in a limited subset of proteins.

The TreeSHAP beeswarm plot revealed distinct contribution patterns among the prioritized proteins (Figure 3C). formimidoyltransferase cyclodeaminase (FTCD), aldolase B, fructose-bisphosphate (ALDOB), betaine-homocysteine S-methyltransferase 2 (BHMT2), complement component (3b/4b) receptor 1 (CR1), LRP2, DCXR, RBP1 and plakophilin 1 (PKP1) were among the highest-ranked features, with mean absolute TreeSHAP values of approximately 0.601, 0.429, 0.322, 0.273, 0.228, 0.200, 0.173, and 0.104, respectively.

A representative TreeSHAP waterfall plot illustrated the local contribution of individual features to the prediction of a single sample (Figure 3D). Starting from the baseline model output, the contributions of the major features cumulatively shifted the prediction toward the final model output.

KEGG pathway enrichment analysis of the 15 prioritized proteins identified several enriched pathways, including one carbon pool by folate, lysosome, histidine metabolism, butanoate metabolism, pentose phosphate pathway, folate transport and metabolism, beta-alanine metabolism, propanoate metabolism, fructose and mannose metabolism, and pentose and glucuronate interconversions (Figure 3F). Among these pathways, one carbon pool by folate showed the strongest enrichment signal.

To confirm the clinical relevance of the 15 core differential proteins, we used the Nephroseq database to analyze their expression correlations with GFR and serum creatinine (Supplementary Figures S3,S4) For GFR: BASP1, RALY, IGF2R, RBP1, RFTN1, and ARL3 showed significant negative correlation. BHMT2, CR1, DCXR, ASAH1, FTCD, LRP2, plakophilin 1 (PKP1), ABAT, and aldolase B, fructose-bisphosphate (ALDOB) exhibited significant positive correlations. For serum creatinine level: BASP1, CR1, IGF2R, PKP1, RBP1, and RFTN1 expression had significant positive correlations. BHMT2, DCXR, ASAH1, FTCD, ARL3, RALY, LRP2, ABAT, and ALDOB expression showed significant negative correlations. We analyzed the transcriptional changes of 15 core differential proteins through the renal sequencing database, and these mRNA level changes were consistent with the proteomic profiling results (Supplementary Figure S5). These results suggested that the 15 core proteins may be related to the progression of DN.

### 3.3 Single-cell mapping identifies podocyte-specific RBP1 as a key candidate target

We performed scRNA-seq analysis using the GSE209781 dataset. After quality control, a total of 64,595 cells were obtained, among which 30,293 cells were from normal human kidney tissues and 34,302 cells from kidney tissues of patients with DN. Subsequently, strict quality control yielded a set of 51,315 single cells, with 24,736 and 26,579 cells derived from kidney tissues of patients in normal and DN group, respectively. Through UMAP clustering analysis, 33 distinct cell clusters were identified (Figure 4A), leading to the recognition of 13 cell types: proximal tubule cells, distal convoluted tubule-loop of henle cells (DCT-LOH), podocytes, glomerular endothelial cells (GEnC), glomerular mesangial cells (GMC), fibroblasts, B cells, macrophages, Mast cells, neutrophils, T cells, T cell and natural killer cell-related population (T_NK), and other cells (Figure 4B). The distribution proportions of each cell type across individual kidney tissues were displayed in Figure 4C.

**FIGURE 4:**
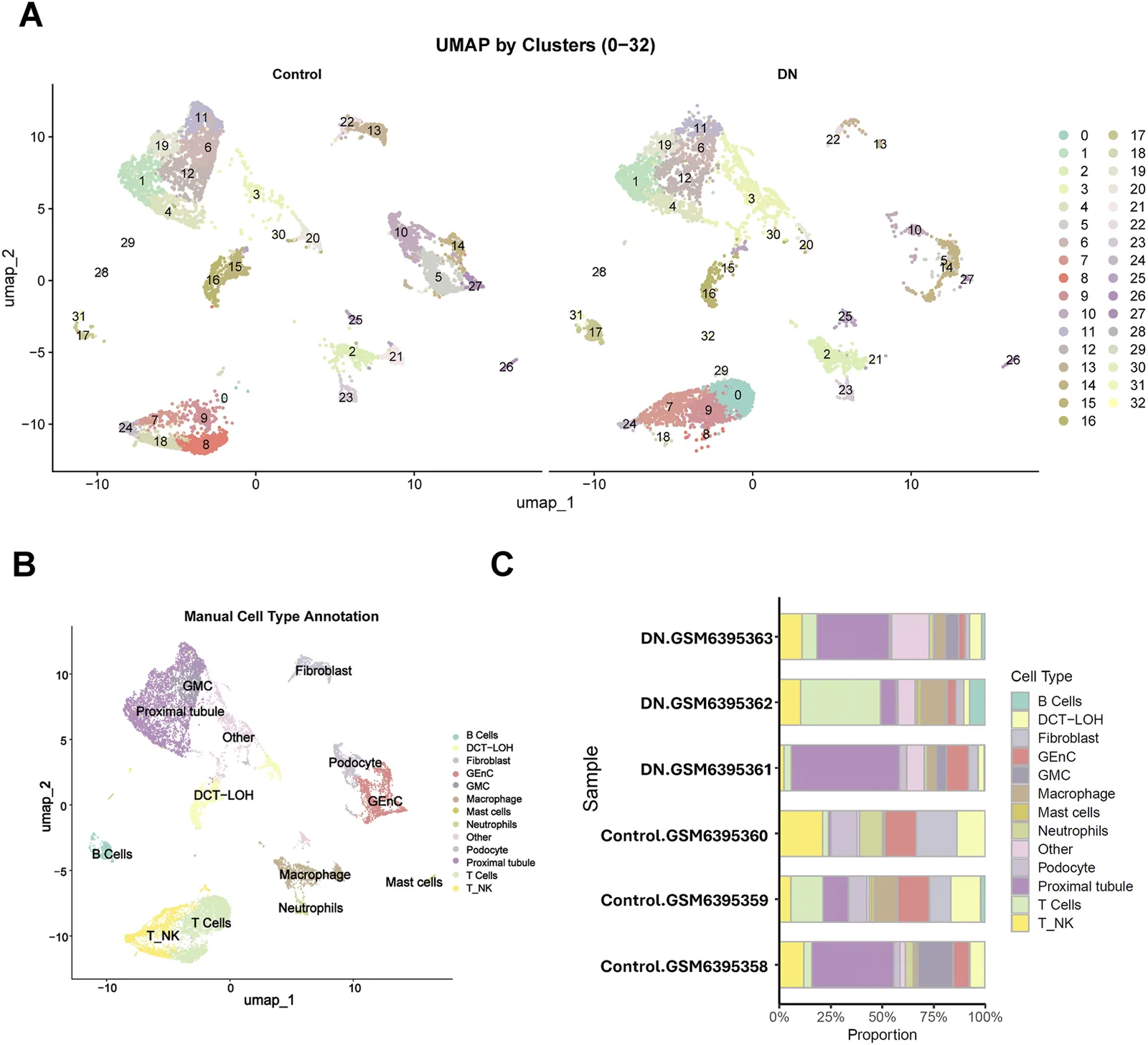
Single-cell RNA sequencing (scRNA-seq) of GSE209781 dataset. **(A)** Clustering analysis identified 33 distinct cell clusters, which were visualized using UMAP dimensionality reduction. **(B)** Annotation revealed 13 cell types: Proximal tubule cells, DCT-LOH (Distal Convoluted Tubule-Loop of Henle cells), Podocytes, GEnC (Glomerular Endothelial Cells), GMC (Glomerular Mesangial Cells), Fibroblasts, B cells, Macrophages, Mast Cells, Neutrophils, T Cells, T_NK (T Cell and Natural Killer Cell-Related Population), and Other Cells. **(C)** Stacked bar plot illustrating the proportional distribution of each cell type in kidney tissue of control and DN patients.

Based on the 15 core differentially expressed proteins identified via machine learning in the previous results, we further analyzed their cell type-specific distribution in human kidney tissues using scRNA-seq. The results revealed that the transcript levels of four proteins (DCXR, IGF2R, LRP2, and RBP1) were significantly and specifically upregulated in podocytes of patients with DN (Supplementary Figure S6).

Among these, RBP1 exhibited the most prominent cell-type specificity: its expression was restricted to GEnC, fibroblasts, and podocytes, with the most significant upregulation observed in DN podocytes (Figures 5A,B). To verify the reliability of targets screened by machine learning and evaluate the diagnostic value of RBP1 for DN, we generated a ROC curve using the independent human DN glomerular transcriptome dataset (GSE96804).

**FIGURE 5:**
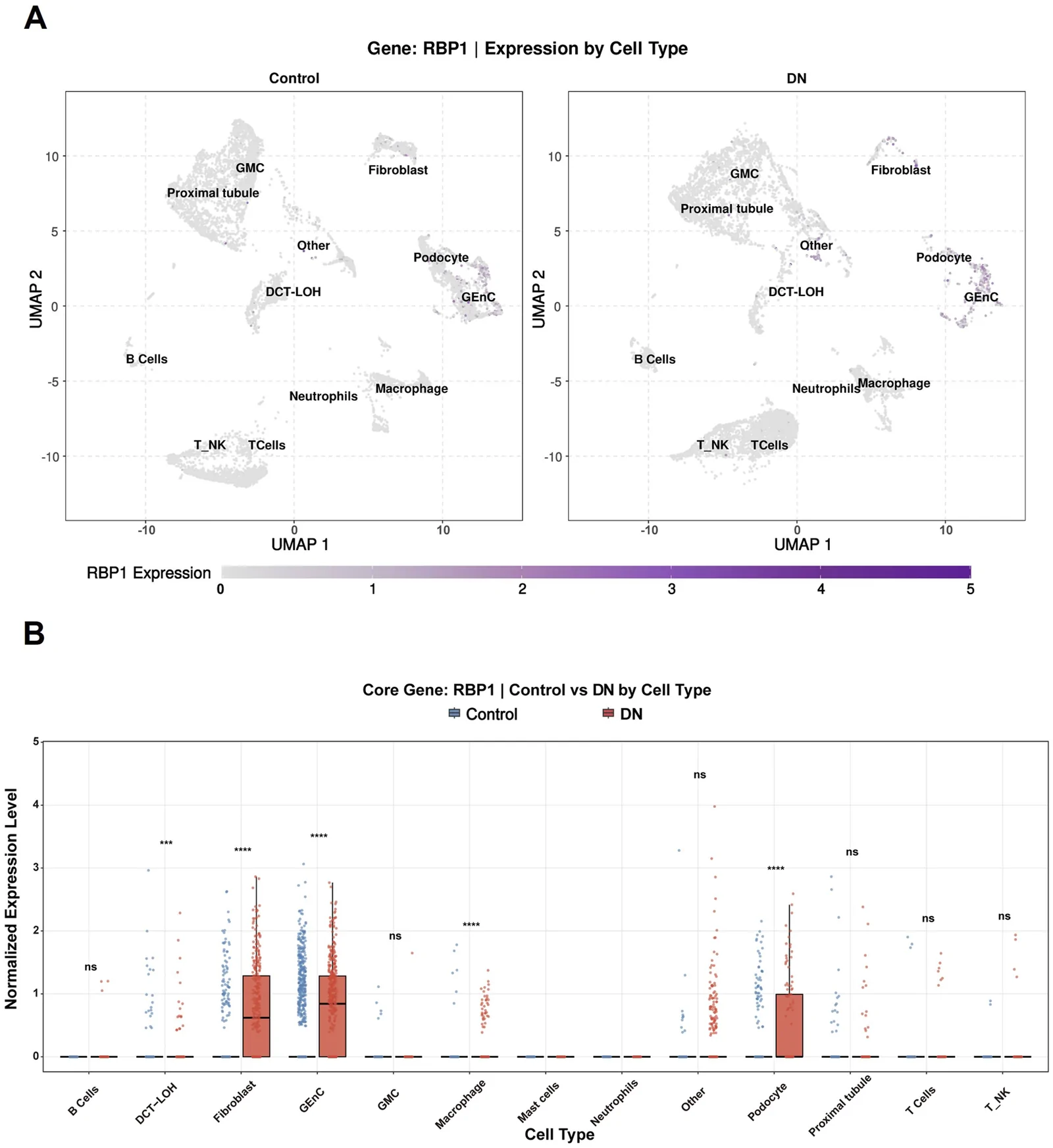
Cell-specific expression of RBP1 in DN renal tissues. **(A)** UMAP plots showing the distribution of RBP1 expression in renal single-cell transcriptomes of the control and DN patients, and **(B)** boxplots comparing RBP1 expression levels across different cell types between the two groups. ***P < 0.001, ****P < 0.0001; ns, not significant.

The area under the curve (AUC) of RBP1 for discriminating DN from control human kidney samples was 0.77 (95% CI: 0.653–0.886), indicating that RBP1 has favorable diagnostic performance for DN (Supplementary Figure S7).

### 3.4 RBP1 upregulation is associated with enhanced podocyte-macrophage communication and M2 macrophage enrichment in DN

To characterize intercellular communication in DN, we constructed cell-cell interaction networks using scRNA-seq. Compared with control kidney tissues, DN samples exhibited significantly enhanced signaling crosstalk between podocytes, GEnC, fibroblasts and macrophages. Notably, podocytes showed the most prominent increase in connectivity with macrophages, identifying podocyte-macrophage communication as a key pathogenic feature of DN (Figure 6A). To further dissect the immune landscape underlying this enhanced intercellular crosstalk, we systematically compared the proportions of infiltrating immune cell subsets between DN and control patients. The data were derived from the GSE96804 and GSE111154, Control: n = 24; DN: n = 45. The scRNA-seq dataset (GSE209781) was not adopted for this quantitative comparison: although it supported cell-type-specific expression and cell-cell communication analysis at single-cell resolution, it only included three control and 3 DN human kidney samples. Given the high inter-individual heterogeneity of immune cell infiltration profiles, the larger bulk RNA-seq cohort was therefore used to yield more reliable conclusions. Analysis revealed that kidney tissues of DN exhibited a distinct immune cell infiltration profile, with significantly elevated fractions of monocytes, M1 macrophages, and particularly M2 macrophages, alongside altered distribution of several adaptive immune cell subsets (Figure 6B). Notably, the accumulation of the M2 subset was much more pronounced, indicating a prominent predominance of M2 macrophage infiltration within the DN renal microenvironment.

**FIGURE 6:**
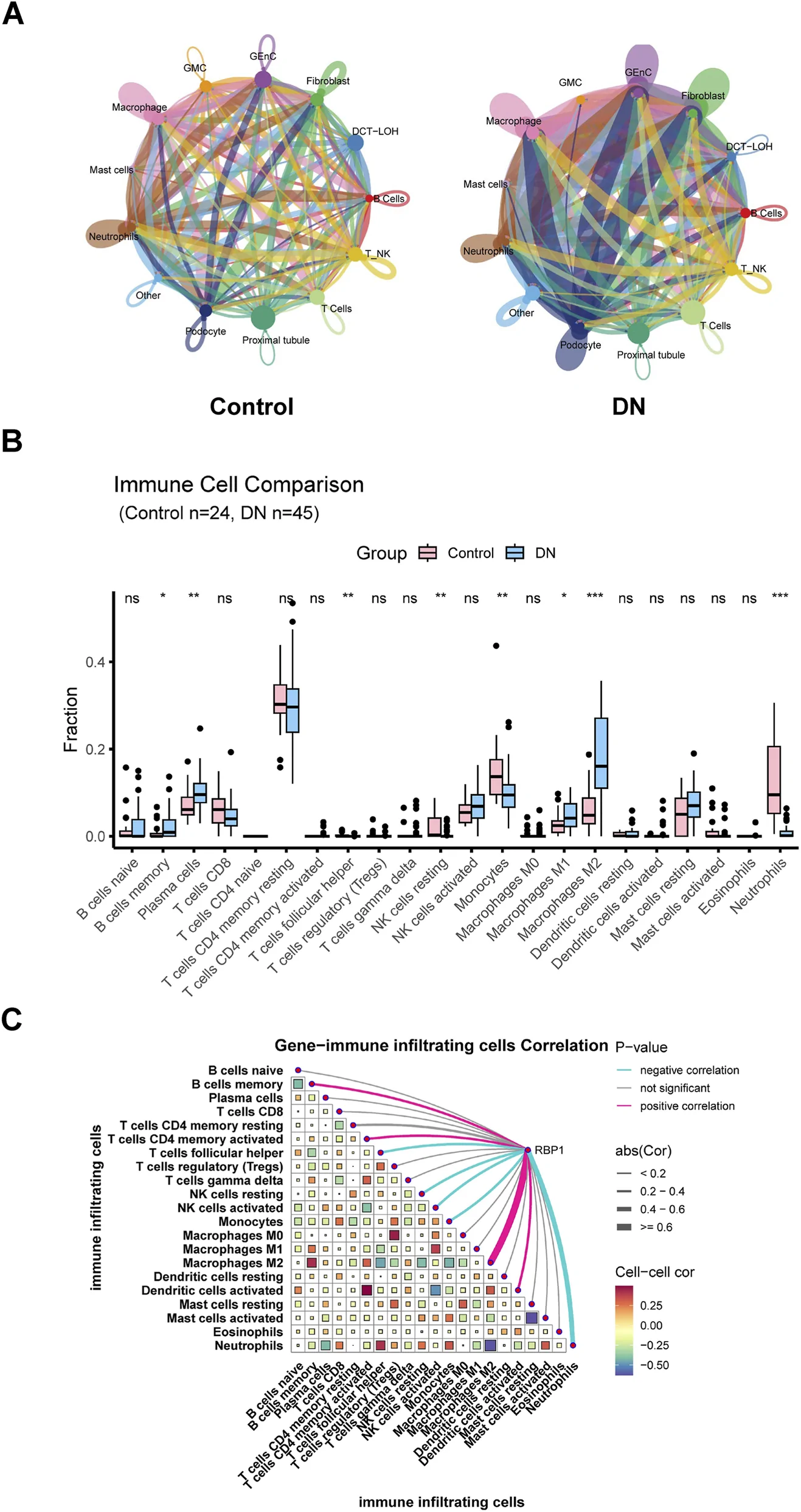
Association of RBP1 with cell-cell crosstalk and immune microenvironment remodeling in DN. **(A)** Single-cell communication networks in renal tissues from control and DN patients. Nodes denote distinct cell types, and edges represent intercellular communication interactions. Edge color and thickness reflect the type and strength of interactions. The data are derived from the single-cell dataset GSE209781. **(B)** Boxplot comparison of immune cell infiltration proportions between the control and DN patients. *P < 0.05, **P < 0.01, ***P < 0.001; ns, not significant. The data are derived from the GSE96804 and GSE111154 datasets. **(C)** Correlation analysis between RBP1 expression and infiltrating immune cells in renal tissue. The data are derived from the GSE96804 and GSE111154.

To explore the potential link between podocyte-specific RBP1 upregulation and immune cell infiltration, we performed correlation analysis between expression level of RBP1 and the fraction of infiltrating immune cells. Notably, RBP1 expression showed a significant positive correlation with M2 macrophage infiltration (Figure 6C), suggesting that podocyte-derived RBP1 may participate in regulating macrophage chemotaxis or polarization during DN progression.

These findings suggested that podocyte-specific upregulated RBP1 may participate in the communication between podocytes and macrophages during DN progression.

### 3.5 RBP1 promoted PA-induced podocyte injury and macrophage chemotaxis

To investigate the potential role of RBP1 in podocyte injury used PA-stimulated human podocytes HPCs as an *in vitro* model.

We first validated the cytotoxic effect of PA on HPCs using CCK-8 assay with gradient concentrations and treatment durations. Compared with the BSA vehicle control, treatment with 50, 100, and 200 μM PA did not cause a significant decrease in HPCs viability, and significant cell death was observed only when the PA concentration reached 300 μM or higher. Time-course analysis showed that cell viability remained unchanged at 12, 24, and 36 h of 100 μM PA treatment relative to the 0 h baseline, while a significant reduction was detected only after 48 h of exposure (Supplementary Figures S8A,B). Western blot analysis showed that PA stimulation significantly reduced nephrin and synaptopodin protein levels in a time-dependent manner compared with the 0 h control group (Figures 7A,B). Concurrently, qRT-PCR quantification showed that pro-inflammatory factors CCL2, IL-6, and TNF-α mRNA levels were significantly upregulated in this cell model (Figures 7C–E). Furthermore, a transwell migration assay showed that the conditioned medium from PA-stimulated HPCs markedly enhanced macrophage chemotaxis over time compared with the 0 h control group (Figure 7F).

**FIGURE 7:**
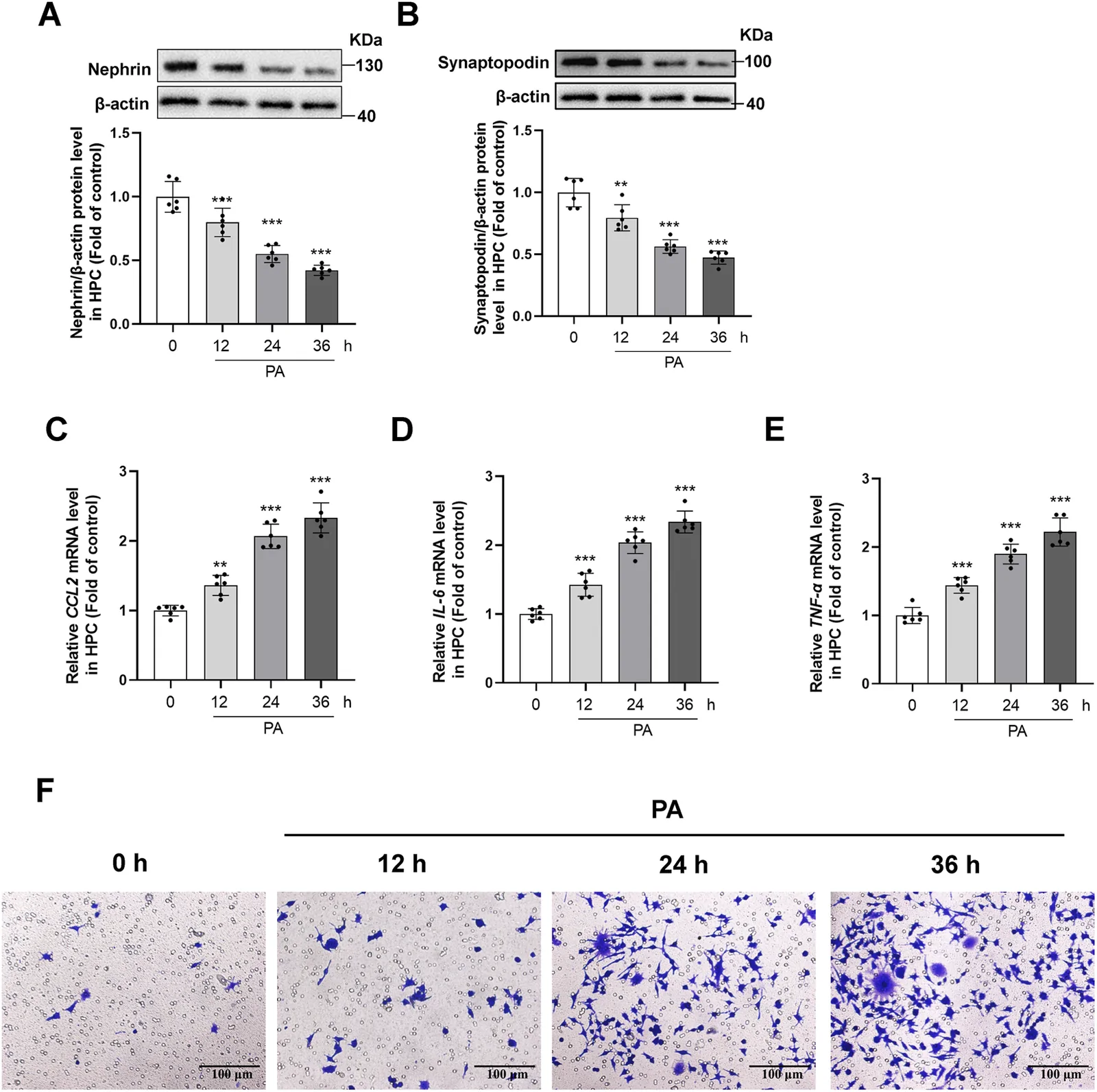
PA induces podocyte injury and pro-inflammatory activation. **(A,B)** The protein levels of nephrin **(A)** and synaptopodin **(B)** in HPCs stimulated with 100 μM PA for 0, 12, 24, and 36 h. The protein levels normalized to β-actin (n = 6). **(C–E)** qRT-PCR quantification of the mRNA expression of pro-inflammatory genes *CCL2* **(C)**, *IL-6* **(D)**, and *TNF-*α **(E)** in PA-stimulated HPCs. The mRNAs were normalized to *β-actin* and presented as fold change relative to the 0 h control group (n = 6). **(F)** Transwell migration assay showing THP-1 macrophage chemotaxis induced by PA-stimulated HPCs (scale bar = 100 μm). All data are presented as mean ± standard deviation (SD). **P < 0.01, ***P < 0.001 compared with the 0 h group.

Stimulation with 100 μM PA time-dependently increased both *RBP1* mRNA levels and RBP1 protein levels in HPCs compared with the 0 h control group (Figures 8A,B). Knockdown of RBP1 using *RBP1* siRNA successfully upregulated the protein expression level of nephrin and synaptopodin in PA-stimulated HPCs compared with the PA-stimulated negative control group (Figures 8C,D).

**FIGURE 8:**
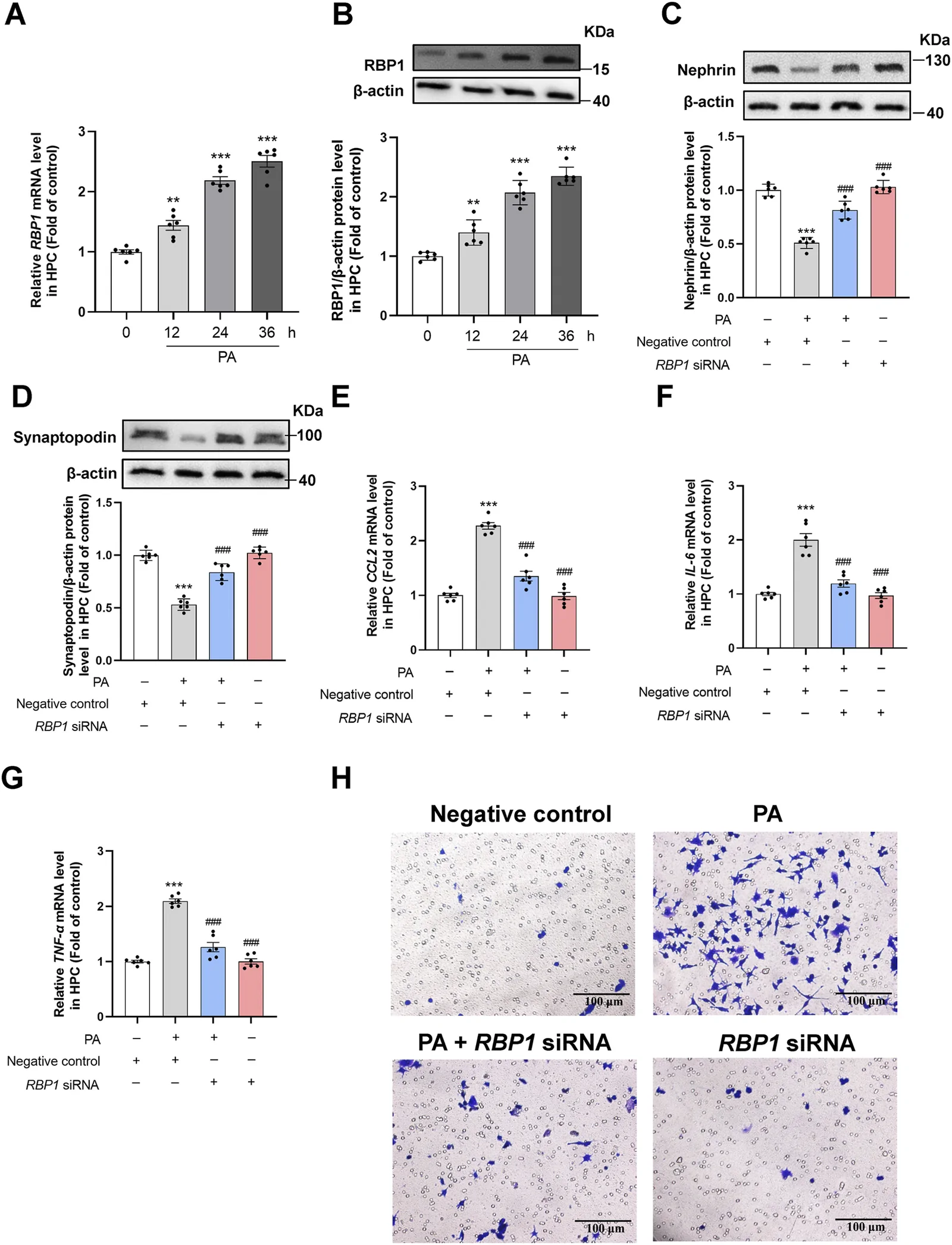
RBP1 upregulation mediates PA-induced podocyte injury and pro-inflammatory responses. **(A)** qRT-PCR quantification of the mRNA expression of RBP1 in HPCs stimulated with 100 μM PA for 0, 12, 24, and 36 h (n = 6). Expression levels are presented as fold change relative to the 0 h control. **(B)** Western blot analysis of RBP1 protein expression in HPCs stimulated with 100 μM PA for 0, 12, 24, and 36 h (n = 6). **(C,D)** Western blot detection of nephrin **(C)** and synaptopodin **(D)** in HPCs, which were stimulated with or without 100 μM PA after being transfected with siRNA-NC or siRNA-*RBP1* (n = 6). **(E–G)** qRT-PCR analysis of pro-inflammatory cytokine mRNA levels, including *CCL2* **(E)**, *IL-6* **(F)**, and *TNF-*α **(G)** in HPCs, which were stimulated with or without 100 μM PA after being transfected with siRNA-NC or siRNA-*RBP1*. Gene expression was normalized to *β-actin* and presented as fold change relative to the negative control group (n = 6). **(H)** Transwell macrophage migration assay. Macrophage migration was induced by conditioned medium collected from HPCs, which were stimulated with or without 100 μM PA after being transfected with siRNA-NC or siRNA-*RBP1*. Scale bar = 100 μm. Data are presented as mean ± standard deviation (SD). **P < 0.01, ***P < 0.001 vs. the 0 h or negative control group; ###*P* < 0.001 vs. the PA group.

Additionally, *RBP1* siRNA intervention significantly suppressed PA-induced upregulation of *CCL2*, *IL-6*, and *TNF-α* mRNA levels compared with the PA-stimulated negative control group (Figures 8E–G). The Transwell migration assay also confirmed that RBP1 knockdown diminished macrophage chemotaxis induced by the conditioned medium of PA-stimulated HPCs (Figure 8H).

Suppression of NF-κB signaling ameliorates podocyte damage, restrains pro-inflammatory cytokine secretion, and reduces macrophage infiltration in the kidney of STZ-induced DKD mice (Li et al., 2025; Sunilkumar et al., 2025). To verify whether RBP1 induced inflammatory responses by activating NF-κB, we detected p-p65, t-p65, and IκBα protein levels. Western blot analysis showed that PA exposure increased p-p65 protein levels and decreased IκBα protein levels compared with the negative control group; however, these changes were significantly reversed by *RBP1* siRNA transfection (Figure 9A). Immunofluorescence staining further confirmed that p65 was mainly retained in the cytoplasm under normal condition, whereas PA stimulation induced prominent nuclear translocation of p65 in HPCs. Knockdown of RBP1 effectively inhibited the PA-induced nuclear accumulation of p65 in HPCs (Figure 9B). To further assess NF-κB activation at the biochemical level, we examined the interaction between IκBα and p65 by Co-IP. PA stimulation significantly reduced the amount of IκBα co-immunoprecipitated with p65 compared with the NC group, indicating enhanced dissociation of the IκBα–p65 complex. In contrast, *RBP1* knockdown markedly restored the association between IκBα and p65 in PA-induced HPCs (Figure 9C). These results indicated that RBP1 promoted PA-induced podocyte injury, pro-inflammatory cytokine secretion and macrophage chemotaxis via activation of NF-κB signaling.

**FIGURE 9:**
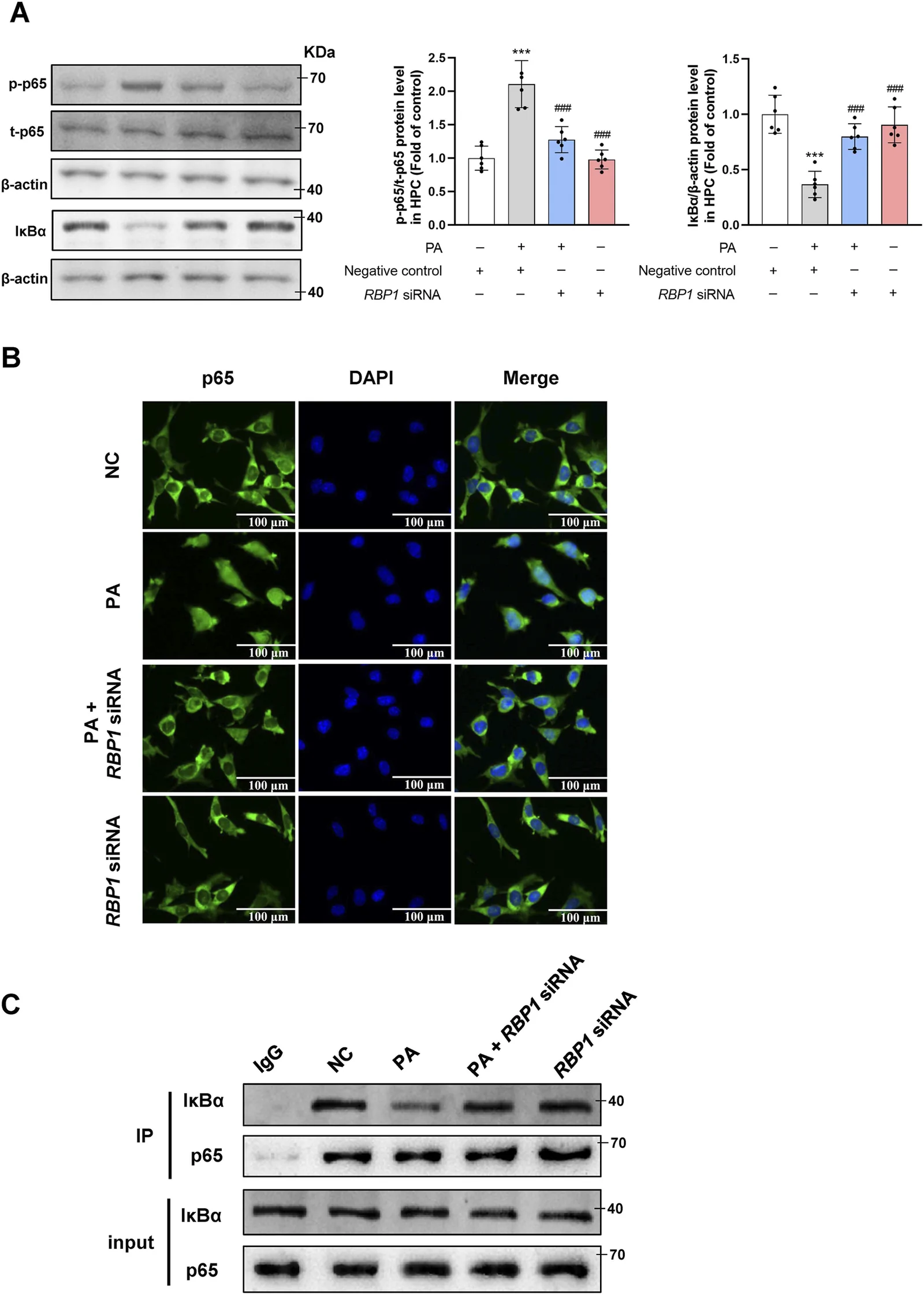
RBP1 knockdown attenuates PA-induced NF-κB signaling activation in podocytes. **(A)** Western blot detection of p-p65 and IκBα in HPCs, which were stimulated with or without 100 μM PA after being transfected with siRNA-NC or siRNA-*RBP1* (n = 6). **(B)** Immunofluorescence staining of p65 (green) and DAPI (blue) to detect the nuclear translocation of p65 in HPCs. Scale bar = 100 μm. Data are presented as mean ± standard deviation (SD). **(C)** Representative Co-IP analysis of the IκBα–p65 interaction in HPCs under the indicated conditions. p65 was immunoprecipitated from cell lysates, and associated IκBα was detected by Western blotting (n = 3). ***P < 0.001 vs. the negative control group; ###*P* < 0.001, vs. the PA group.

To further clarify the effect of podocyte-derived *RBP1* on macrophage polarization, we examined the expression of M1 and M2 polarization markers in THP-1 macrophages. Compared with siNC-CM, PA + siNC-CM significantly increased M1-associated markers *CD80*, *TNF-α*, and *IL-6* mRNA levels. These increases were markedly attenuated by *RBP1* knockdown in podocytes, whereas si*RBP1*-CM alone showed no obvious changes relative to siNC-CM (Supplementary Figures S9A–C).

PA + siNC-CM also markedly increased M2-associated markers *CD163*, *CD206*, and *TGF-β1*, with a more pronounced induction than that observed for the M1 associated markers. These increases were substantially reversed by podocyte *RBP1* knockdown, while si*RBP1*-CM alone remained close to basal levels (Supplementary Figure S9D–F).

### 3.6 Taxifolin targeting RBP1 inhibited NF-κB signal to alleviate podocyte injury and macrophage chemotaxis

To identify potential natural compounds targeting RBP1, we screened the active ingredients of LGZG formula using the Traditional Chinese Medicine Systems Pharmacology Database and Analysis Platform (TCMSP) database. Compounds with oral bioavailability (OB) ≥ 30% and drug-likeness (DL) ≥ 0.18 were included for subsequent molecular docking with RBP1 protein one by one (Table 1). Finally, taxifolin, stellasterol and glycyrol with the lowest binding energies were selected for further MST verification (Figure 10A). MST assays verified that only taxifolin directly bound to RBP1. No specific binding signals were detected for stellasterol or glycyrol under the tested condition (Figure 10B). To test the efficacy of taxifolin, HPCs were co-treated with PA and different concentrations of taxifolin (5, 10, and 20 μM). Taxifolin did not alter PA-induced upregulation of *RBP1* mRNA levels (Figure 10C), it significantly decreased RBP1 protein levels in a dose-dependent manner compared with the PA vehicle group (Figure 10D).

**TABLE 1:** Active ingredients of LGZG formula and their molecular docking binding energy scores with RBP1 protein.

Molecule name	Oral bioavailability (%)	Drug-likeness	Vina score with RBP1(kcal/mol)
Glycyrol	90.78	0.67	−10.1
Taxifolin	57.86	0.27	−9.6
Stellasterol	60.22	0.26	−9.3
(+)-Vestitol	74.66	0.21	−8.9
Beta-sitosterol	36.91	0.75	−8.6
Ent-epicatechin	48.96	0.24	−8.6
Gancaonin-L	68.21	0.35	−8.6
(+)-catechin	54.83	0.27	−8.5
Phaseol	78.77	0.58	−8.5
Licochalcone B	76.76	0.19	−8.4
Liquiritin	65.69	0.74	−8.3
14-Acetyl-12-senecioyl-2E,8Z,10E-atractylentriol	60.31	0.31	−8.2
Formononetin	69.67	0.21	−8.1
α-Amyrin	39.51	0.76	−7.6
3β-acetoxyatractylone	54.07	0.22	−7.5
12-Senecioyl-2E,8E,10E-atractylentriol	62.4	0.22	−7.4
Cerevisterol	47.52	0.32	−7.1
Poricoic acid A	59.13	0.28	−7
8β-ethoxy atractylenolide III	35.95	0.21	−6.8
Dehydrotumulosic acid	36.81	0.35	−6.7
16alpha-hydroxydehydrotrametenolic acid	45.36	0.47	−6.7
Eburicoic acid	35.54	0.45	−6.6

**FIGURE 10:**
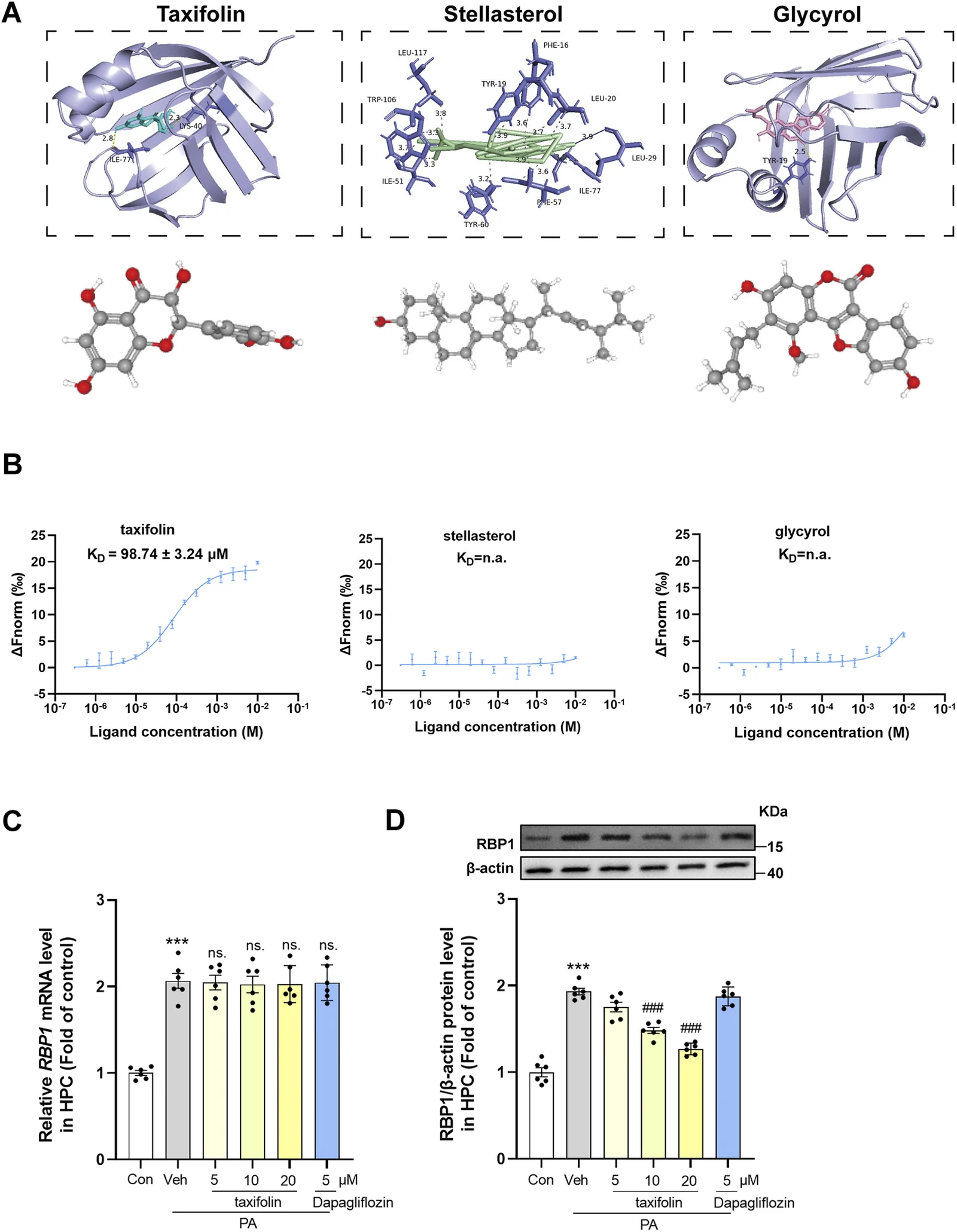
Validation of bioactive compounds of Linggui Zhugan (LGZG) formula targeting RBP1. **(A)** Representative molecular docking models of three selected compounds with RBP1. **(B)** MST validation of the binding affinity between the three compounds and RBP1. **(C,D)** Expression of RBP1 in HPCs treated with 100 μM PA in the presence or absence of taxifolin (5, 10, and 20 μM) or dapagliflozin (5 μM). **(C)** qRT-PCR analysis of *RBP1* mRNA; **(D)** Western blot analysis of RBP1 protein (n = 6). Data are presented as mean ± SD. ***P < 0.001 vs. the control group; ###*P* < 0.001 vs. the PA group; ns, not significant vs. the control group.

Taxifolin (10 and 20 μM) and dapagliflozin (5 μM) significantly alleviated PA-induced reduction of nephrin and synaptopodin protein levels compared with the PA vehicle group (Figure 11A). Taxifolin dose-dependently reduced *CCL2*, *IL-6*, and *TNF-α* mRNA levels in PA-stimulated HPCs compared with the PA vehicle group (Figures 11B–D). The Transwell migration assay further revealed that taxifolin or dapagliflozin could significantly inhibit the enhanced chemotaxis of macrophages induced by PA-stimulated HPCs (Figure 11E).

**FIGURE 11:**
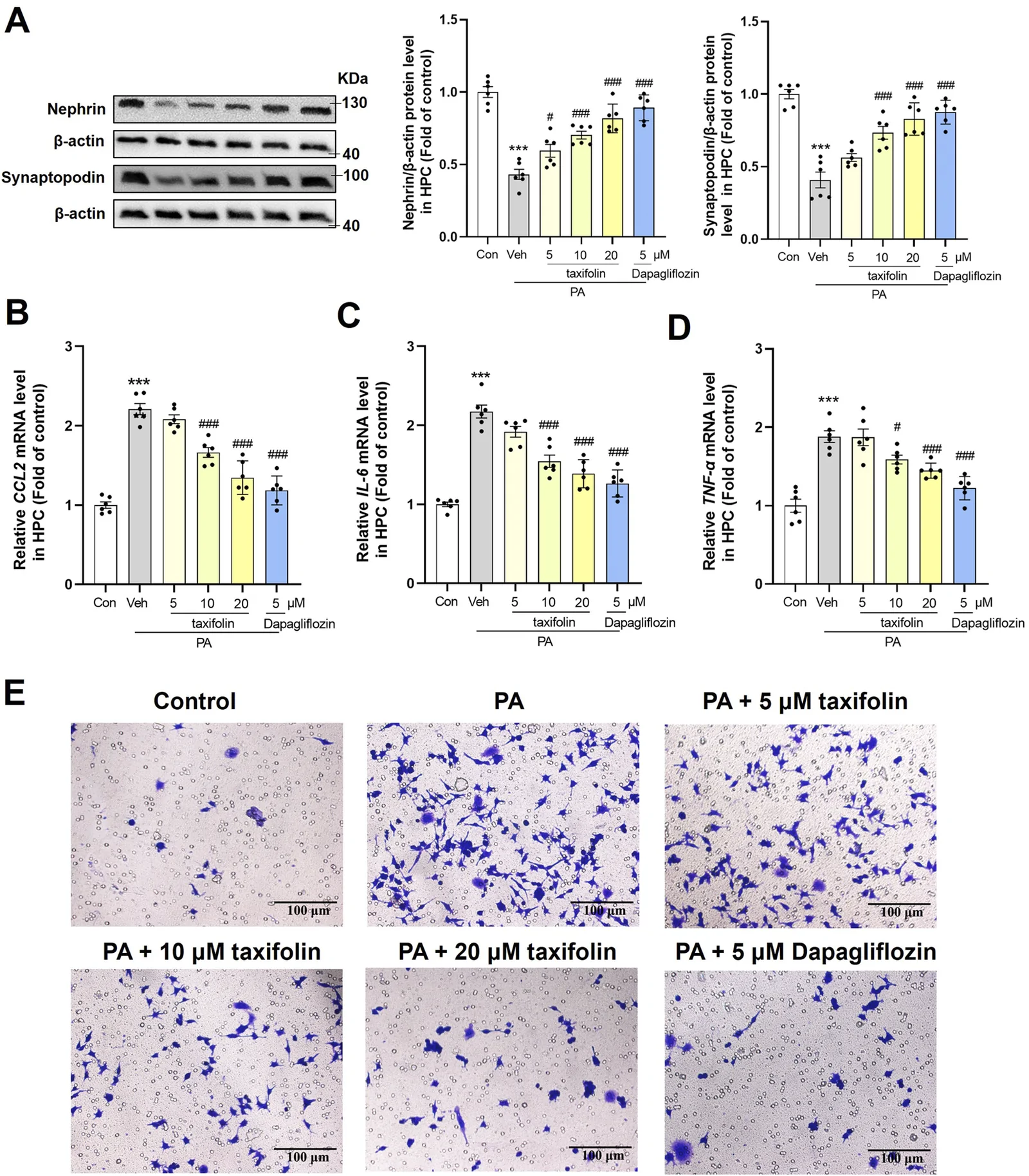
Taxifolin ameliorates PA-induced podocyte injury and pro-inflammatory responses *in vitro*. HPCs were cultured with or without 100 μM PA, taxifolin (5, 10, and 20 μM) or dapagliflozin (5 μM). **(A)** Western blot analysis of podocyte injury markers nephrin and synaptopodin in HPCs (n = 6). **(B–D)** qRT-PCR analysis of pro-inflammatory cytokines *CCL2* **(B)**, *IL-6* **(C)**, and *TNF-*α **(D)** mRNA expression in HPCs (n = 6). **(E)** THP-1 macrophage migration was induced by conditioned medium collected from HPCs, which were cultured with or without 100 μM PA, taxifolin (5, 10, and 20 μM) or dapagliflozin (5 μM). Scale bar = 100 μm. Data are presented as mean ± SD. ***P < 0.001 vs. the control group; #*P* < 0.05, ###*P* < 0.001 vs. the PA group.

Furthermore, taxifolin and dapagliflozin effectively suppressed PA-induced p-p65 phosphorylation and prevented IκBα degradation compared with the PA vehicle group (Figure 12A). Taxifolin dose-dependently attenuated PA-induced nuclear accumulation of p65 in HPCs (Figure 12B). These results suggested that taxifolin targeting RBP1 inhibited NF-κB signaling to alleviate podocyte injury, pro-inflammatory activation and macrophage chemotaxis.

**FIGURE 12:**
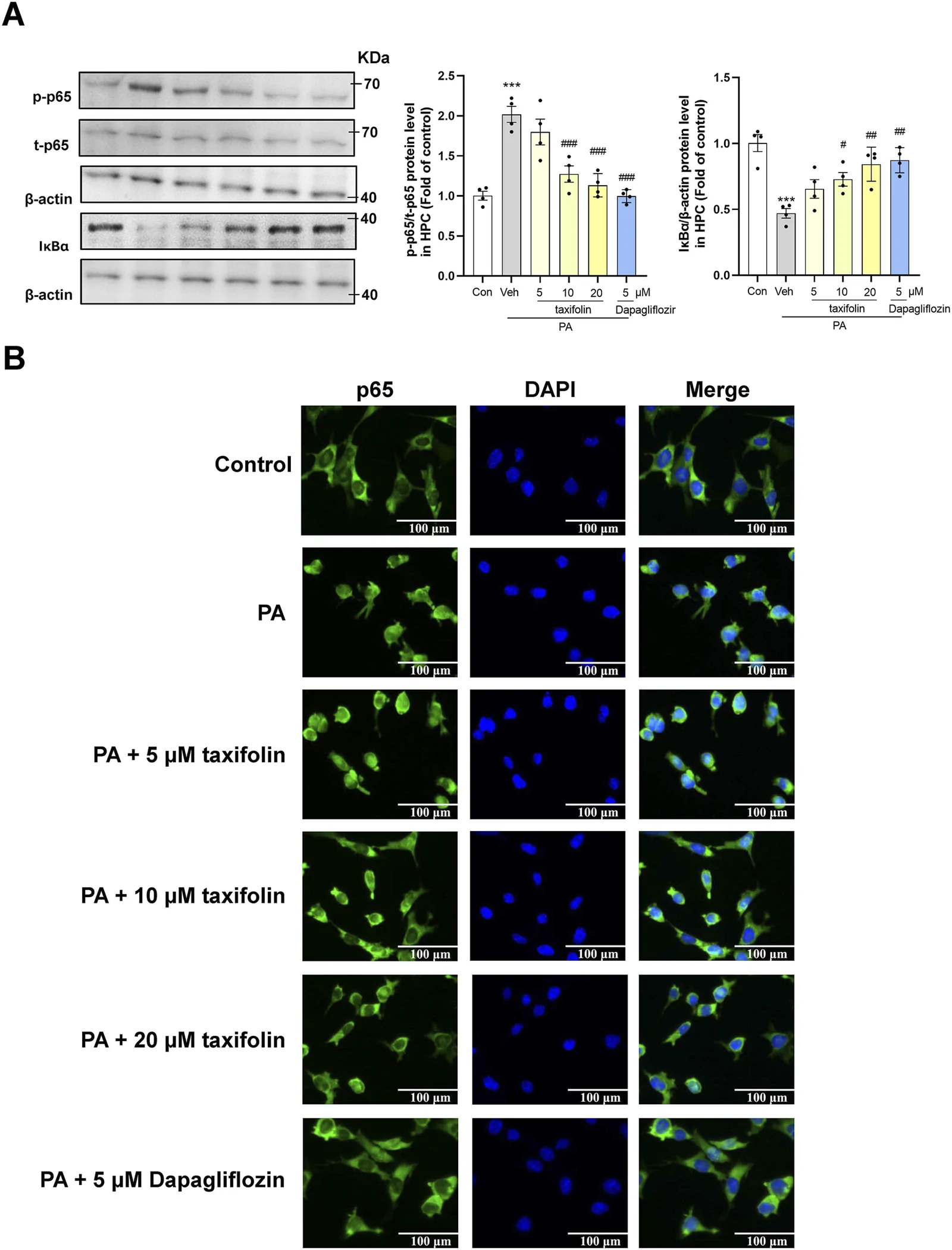
Taxifolin ameliorates PA-induced NF-κB signaling activation in podocytes. **(A)** Western blot detection of p-p65 and IκBα in HPCs, which were cultured with or without 100 μM PA, taxifolin (5, 10, and 20 μM) and dapagliflozin (5 μM) (n = 4). **(B)** Immunofluorescence staining of p65 (green) and DAPI (blue) to detect the nuclear translocation of p65 in HPCs. Scale bar = 100 μm. Data are presented as mean ± standard deviation (SD). ***P < 0.001 vs. the control group; #*P* < 0.05, ##*P* < 0.01, ###*P* < 0.001 vs. the PA group.

Given that our previous bioinformatics findings were based on human clinical datasets, we performed all core *in vitro* functional experiments using human podocytes HPCs and human THP-1 macrophages to ensure translational relevance and consistency across the study. Parallel validation experiments were also conducted in mouse MPC5 podocytes or RAW264.7 macrophages, and the corresponding results were provided in the Supplementary Figures S10–S12.

## 4 Discussion

In this study, we innovatively combined proteomics with single-cell transcriptomic analyses using kidney tissues from DN patients and further verified that RBP1 promotes inflammation and podocyte injury in PA-stimulated podocytes. This screening strategy from renal proteomic profiling to cellular localization reduced the blindness of target verification. Cell–cell communication analysis further revealed that the crosstalk among podocytes, GEnC and fibroblasts with macrophages was remarkably enhanced in DN patients, among which the communication between podocyte and macrophage showed the most obvious change.

The regularized XGBoost model and repeated out-of-fold exact TreeSHAP analysis showed that the predictive information was concentrated within a limited subset of proteins, with the top eight features accounting for 80% of the cumulative mean absolute SHAP contribution and all Top15 proteins showing non-zero contributions. However, these SHAP values primarily reflected each feature’s contribution to model output rather than its standalone diagnostic capacity.

The relatively small sample size compared with the high-dimensional proteomic candidate space remains an important limitation of the machine-learning analysis in this study. Although model complexity was constrained through shallow trees, shrinkage, row and feature subsampling, L1/L2 regularization, repeated cross-validation, and out-of-fold feature attribution, these procedures cannot completely eliminate the risk of overfitting or optimistic performance estimates. Therefore, the predictive performance of the model should be interpreted cautiously and requires validation in larger independent proteomic cohorts.

Among the top 15 core proteins selected through machine learning, RBP1 is also expressed in GEnC and fibroblasts cells in DN patients. Although RBP1 was not exclusively expressed in podocytes, several observations supported our attention to focus on podocytes. Firstly, podocyte injury is a critical initiating event in DN patients (Molitch et al., 2015). More importantly, cell-cell communication analysis identified podocytes as a major interaction hub linking glomerular resident cells and infiltrating macrophages. RBP1 was not among the strongest loading proteins defining the global PCA separation, ranking 12th in PC1 and 37th in PC2. This indicated that RBP1 should not be interpreted as a dominant determinant of the overall proteomic variance in DN (Supplementary Figure S1).

RBP1 is mainly responsible for the intracellular storage and metabolism of retinol (Chou et al., 2012). Our study revealed that the upregulation of RBP1 may serve as a biomarker of DN, as well as act as a key driving factor for podocyte injury in DN.

It is reported that M1 macrophages primarily contribute to inflammatory injury during the early stages of DN by producing pro-inflammatory mediators and aggravating renal damage (Yan et al., 2023). However, M2 macrophages have more complex roles (Tang et al., 2019). The persistent accumulation of M2 macrophages during chronic DN may contribute to tissue remodeling and renal fibrosis (Tang et al., 2019; Calle and Hotter, 2020). As DN progresses, a shift toward an M2 phenotype may occur as a compensatory response to tissue injury (Youssef et al., 2024). In human DN, glomerular CD163-positive macrophages are positively associated with DN class, interstitial fibrosis and tubular atrophy, and glomerulosclerosis (Klessens et al., 2017). Our analysis of the patient-derived GSE96804 and GSE111154 cohorts showed the increased proportions of both M1 and M2 macrophages, with a relative predominance of the M2 phenotype, which likely reflected the chronic pathological state of the study populations. In this study, our data demonstrated that podocyte RBP1 promoted both M1 inflammatory activation and M2 phenotypic shift. Knockdown of podocyte RBP1 effectively reversed abnormal macrophage polarization, further supporting the value of RBP1 as a potential therapeutic target for DN.

Taxifolin, a flavonoid, has gained the attention for its large pharmacological actions (Liu et al., 2023). Taxifolin is reported to exert renoprotective effects against DN in high-fat diet/STZ-induced DN rats, as evidenced by attenuated urinary microalbumin excretion, improved hyperglycemia and lipid metabolism disorders, and mitigated renal histopathological lesions (Ding et al., 2018). Taxifolin inhibits mitogen-activated protein kinase (MAPK) pathway phosphorylation and decreases the expression of pro-inflammatory mediators including TNF-α, inducible nitric oxide synthase and cyclooxygenase-2 in a lipopolysaccharide (LPS)-induced macrophage inflammation model *in vitro* (Zhang et al., 2022). In this study, we found that taxifolin could directly bind to RBP1, subsequent functional experiments confirmed that taxifolin exerted dose-dependent protective effects against PA-induced podocyte injury, inflammatory response and macrophage chemotaxis, indicating that taxifolin may serve as a promising natural compound for DN treatment by targeting RBP1.

Nevertheless, several limitations still exist in our study. All functional verifications were performed merely in *in vitro* cell lines without *in vivo* validation in DN animal models; single-cell transcriptome analysis relied on public datasets, which may be affected by batch effect and sample heterogeneity. Future work will focus on constructing podocyte-specific RBP1 knockout DN mice to verify its *in vivo* function as well as the renoprotective effect of taxifolin, exploring the molecular interaction mechanism between RBP1 and downstream inflammatory signaling molecules, investigating whether taxifolin modulates this podocyte–macrophage regulatory axis.

## 5 Conclusion

In this study, by integrating multi-omics and machine learning, we identified RBP1 as a novel podocyte-specific driver of podocyte injury and macrophage chemotaxis associated with DN. Experiments *in vitro* showed that PA caused the upregulation of RBP1 protein expression in podocytes, leading to podocyte injury, inflammation and macrophage chemotaxis. We also found that taxifolin, a bioactive compound derived from the LGZG formula, could directly bind to RBP1 to alleviate PA-induced podocyte injury and macrophage chemotaxis. These findings identified RBP1 as a novel therapeutic target that may promote podocyte injury in DN and provided taxifolin as a potential therapeutic natural compound.

## Data Availability

The datasets presented in this study can be found in online repositories. The names of the repository/repositories and accession number(s) can be found in the article/Supplementary Material.
