# Supplementary material for: Taxifolin alleviates podocyte injury and macrophage chemotaxis associated with diabetic nephropathy by directly targeting RBP1 identified via multi-omics and machine learning
Source: Front Pharmacol. 2026 Oct 12;17:1933167. doi: 10.3389/fphar.2026.1933167 (PMC13696303; doi:10.3389/fphar.2026.1933167)
Supplement: Supplementary file 1 [file Supplementaryfile1.docx]

## 1.1 Supplementary Figures

**Supplementary Figure 1**. **Top 20 loading proteins for PC1 and PC2.** Bar length indicates the loading weight of each protein on the corresponding principal component. Blue bars represent negative loading weights on PC1, and orange bars represent positive loading weights.

**Supplementary Figure 2. Confusion matrices of eXtreme Gradient Boosting (XGB) model on the independent test set.** The x-axis indicates the true group labels, including the normal control group (Control) and diabetic nephropathy group (DN); the y-axis indicates the group labels predicted by the models. The color gradient represents the number of samples in each corresponding category.

**Supplementary Figure 3. Correlation scatter plots between core differential proteins and serum creatinine levels**. (A–O) Each panel shows the relationship between log₂-transformed expression of the labeled protein and serum creatinine level. All data are retrieved from the nephroseq database.

**Supplementary Figure 4. Correlation scatter plots between core differential proteins and glomerular filtration rate (GFR).** (A–O) Each panel depicts the association between log₂-transformed expression of the labeled protein and log₂-transformed GFR. All data are retrieved from the nephroseq database.

**Supplementary Figure 5.** The transcriptional (mRNA) levels of the key differential proteins screened by machine learning in the kidneys of the normal group and the diabetic nephropathy patient group are verified using the nephroseq database.

**Supplementary Figure 6. Cell-type-specific transcriptional profiles of the top 15 core candidate genes in control and DN human kidney tissues.** Box plots illustrate normalized gene expression across multiple kidney cell subtypes. * *P* < 0.05; ** *P* < 0.01; *** *P* < 0.001, **** *P* < 0.0001; ns, no significant difference.

**Supplementary Figure 7.** ROC curve of RBP1 for diabetic nephropathy diagnosis. The AUC is 0.77 (95% CI: 0.653–0.886), based on the GSE96804 dataset.

**Supplementary Figure 8. Cell viability of HPCs under PA stimulation evaluated by CCK-8 assay.** (A) Concentration-dependent cytotoxicity of PA in HPCs. Cells were treated with gradient concentrations of PA (50, 100, 200, 300, and 500 μM) for 24 h, with BSA-containing medium as the vehicle control. (B) Time-dependent cytotoxicity of 100 μM PA in HPCs. Cells were harvested for viability detection at 0, 12, 24, 36, 48, and 72 h after PA stimulation. Cell viability was calculated as the percentage relative to the control group. Data are presented as mean ± SD (n = 6). ***P* < 0.01, ****P* < 0.001 vs. BSA or 0 h group.

**Supplementary Figure 9. Podocyte *RBP1* modulates M1- and M2-associated marker expression in human THP-1 macrophages under lipotoxic conditions.** Human THP-1 monocytes were differentiated into M0 macrophages with 100 nM PMA for 24 h and subsequently treated with conditioned medium (CM) derived from differently treated HPCs for another 24 h. The mRNA levels of M1- and M2-associated macrophage markers were measured by qRT-PCR. (A–C) M1-associated markers: *CD80* (A), *TNF-α* (B), and *IL-6* (C). (D–F) M2-associated markers: *CD163* (D), *CD206* (E), and *TGF-β1* (F). The CM groups were: siNC-CM, CM from BSA-treated HPCs transfected with siRNA-NC; PA+siNC-CM, CM from PA-treated HPCs transfected with siRNA-NC; PA+si*RBP1*-CM, CM from PA-treated HPCs transfected with siRNA-*RBP1*; and si*RBP1*-CM, CM from BSA-treated HPCs transfected with siRNA-*RBP1*. Data are presented as mean ± SD (n = 6). ****P* < 0.001 vs. siNC-CM; ###*P* < 0.001 vs. PA+siNC-CM.

**Supplementary Figure 10. PA induces MPC5 podocyte injury and pro-inflammatory activation.**

(A) Concentration-dependent cytotoxicity of PA in MPC5. Cells were treated with gradient concentrations of PA (50, 100, 200, 300, and 500 μM) for 24 h, with BSA-containing medium as the vehicle control. (B) Time-dependent cytotoxicity of 100 μM PA in MPC5. Cells were harvested for viability detection at 0, 12, 24, 36, 48, and 72 h after PA stimulation. Cell viability was calculated as the percentage relative to the control group (C) The protein levels of nephrin and synaptopodin (D) in MPC5 stimulated with 100 μM PA for 0, 12, 24, and 36 h, β-actin was used as the control (n = 6). (E-G) qRT-PCR quantification of the mRNA expression of pro-inflammatory genes *Ccl2* (E), *Il-6* (F), and *Tnf-α* (G) in PA-stimulated MPC5. Gene expression was normalized to *β-actin* and presented as fold change relative to the 0 h control group (n = 6). (H) Transwell migration assay showing macrophage recruitment induced by PA-stimulated MPC5 (scale bar = 100 μm). All data are presented as mean ± standard deviation (SD). * *P* < 0.05, ** *P* < 0.01, *** *P* < 0.001 compared with the 0 h control group.

**Supplementary Figure 11. RBP1 upregulation mediates PA-induced MPC5 podocyte injury and pro-inflammatory responses.** (A) qRT-PCR quantification of the mRNA expression of RBP1 in MPC5 stimulated with 100 μM PA for 0, 12, 24, and 36 h (n = 6). Expression levels are presented as fold change relative to the 0 h control. (B) Western blot analysis of RBP1 protein expression in MPC5 stimulated with PA (n = 6). (C-D) Western blot detection of synaptopodin (C) and Nephrin (D) in MPC5, which was stimulated with or without 100 μM PA after being transfected with siRNA-NC or siRNA-*Rbp1* (n = 6). (E-G) qRT-PCR analysis of pro-inflammatory cytokine mRNA levels, including *Ccl2* (E), *Il-6* (F), and *Tnf-α* (G) in MPC5, which was stimulated with or without 100 μM PA after being transfected with siRNA-NC or siRNA-*Rbp1*. Gene expression was normalized to *β-actin* and presented as fold change relative to the negative control group (n = 6). (H) Transwell macrophage migration assay. RAW264.7 macrophage migration was induced by conditioned medium collected from MPC5, which was stimulated with or without 100 μM PA after being transfected with siRNA-NC or siRNA-*Rbp1*. Scale bar = 100 μm. Data are presented as mean ± standard deviation (SD). ** *P* < 0.01, *** *P* < 0.001 vs. the 0 h or negative control group; ### *P* < 0.001, vs. the PA group.

**Supplementary Figure 12. Taxifolin ameliorates PA-induced MPC5 podocyte injury and pro-inflammatory responses *in vitro*.** (A) qRT-PCR analysis of Rbp1 and western blot analysis (B) of RBP1 in MPC5, which was stimulated with 100 μM PA as well as taxifolin (5, 10, and 20 μM) or not (n = 6). (C) MPC5 was cultured with or without 100 μM PA, taxifolin (5, 10, and 20 μM) and dapagliflozin (5 μM). Western blot analysis of podocyte injury markers nephrin and synaptopodin in MPC5 (n = 6). (D-F) MPC5 was cultured with or without 100 μM PA, taxifolin (5, 10, and 20 μM) and dapagliflozin (5 μM). qRT-PCR analysis of pro-inflammatory cytokines *Ccl2* (D), *Il-6* (E), and *Tnf-α* (F) mRNA expression in MPC5 (n = 6). (G) RAW264.7 macrophage migration was induced by conditioned medium collected from MPC5, which was cultured with or without 100 μM PA, taxifolin (5, 10, and 20 μM) and dapagliflozin (5 μM). Scale bar = 100 μm. Data are presented as mean ± SD. *** *P* < 0.001 vs. the control group; # *P* < 0.05, ## *P* < 0.01, ### *P* < 0.001 vs. the PA group.

## 1.2 Supplementary Tables

Supplementary Table 1. Cell type annotation genes.

| Cell_Type | Marker Genes |
| --- | --- |
| T_NK | NKG7, NCAM1, KLRF1 |
| T cells | CD3D, KLRB1 |
| B cells | CD79A, CD79B |
| Neutrophils | S100A9, S100A8 |
| Mast cells | TPSAB1, CPA3 |
| Macrophage | C1QB, C1QA, CD68 |
| Proximal tubule | LRP2, SLC34A1 |
| DCT-LOH | SLC12A1, SLC12A3 |
| Distal Tubule | WNK1, CALB1 |
| CD-PC | AQP2, AQP3 |
| CD-IC | ATP6V1G3, CA2 |
| Fibroblast | COL1A2, DCN |
| GEnC | EMCN, VWF |
| GMC | MCAM, PDGFRB |
| Podocyte | PODXL, SYNPO |

Supplementary Table 2. The primer sequences (h:homo sapiens; m:mouse).

| **ID** | **Sense primer (5′→ 3′)** | **Antisense primer (5′→ 3′)** |
| --- | --- | --- |
| h-*CCL2* | CAGCCAGATGCAATCAATGCC | TGGAATCCTGAACCCACTTCT |
| h-*IL-6* | CTGGCTTGTTCCTCACTACTC | ATCATCACTGGTCTTTTGGAG |
| h-*TNF-α* | CCATCAGAGGGCCTGTACCTCATCT | ACAGGGCAATGATCCCAAAGTAGAC |
| h-*CD206* | CCATCGAGGAAGAGGTTCGG | GGTGGGTTACTCCTTCTGCC |
| h-*CD163* | CCAGAAGGAACTTGTAGCCACAG | CAGGCACCAAGCGTTTTGAGCT |
| h-*CD80* | CACCTTGCCCTTTACGTATCT | CTACTTCTGTGCCCACCATATT |
| h-*TGFB1* | GGCCAGATCCTGTCCAAGC | GTGGGTTTCCACCATTAGCAC |
| h-*β-actin* | CATGTACGTTGCTATCCAGGC | CTCCTTAATGTCACGCACGAT |
| m-*Ccl2* | TTAAAAACCTGGATCGGAACCAA | GCATTAGCTTCAGATTTACGGGT |
| m-*Il-6* | ACCAGAGGAAATTTTCAATAGGC | TGATGCACTTGCAGAAAACA |
| m-*Tnf-α* | AGGGTCTGGGCCATAGAACT | CCACCACGCTCTTCTGTCTAC |
| m-*β-actin* | AAGTGCTTCTAGGCGGACTGTT | TTTTCTGCGCAAGTTAGGTTTTG |

Supplementary Table 3. Results of permutational multivariate ANOVA (PERMANOVA)

| **Source** | **Df** | **Sum of squares** | **R²** | **F value** | **P value** |
| --- | --- | --- | --- | --- | --- |
| Model | 1 | 635 104 | 0.1576 | 14.407 | 0.001*** |
| Residual | 77 | 3 394 358 | 0.8424 | — | — |
| Total | 78 | 4 029 462 | 1.0000 | — | — |

Table note. PERMANOVA was performed based on Euclidean distance with 999 permutations to test the global difference in proteomic profiles between the control and DN groups. *** *P* < 0.001.

Supplementary Table 4. Classification performance metrics of XGB models on the independent test set.

| ModelName | MethodID | Threshold | Accuracy | Sensitivity | Specificity | Precision | Recall | F1 |
| --- | --- | --- | --- | --- | --- | --- | --- | --- |
| XGB | xgbTree | 0.5 | 0.863636 | 0.933333 | 0.714286 | 0.875 | 0.933333 | 0.903226 |

Table note. The classification threshold was set at 0.5, and diabetic nephropathy (DN) was defined as the positive class. Accuracy: overall prediction accuracy; Sensitivity: true positive rate; Specificity: true negative rate; Precision: positive predictive value; Recall: true positive rate; F1: F1-score.
